# STAT3 acts through pre-existing nucleosome-depleted regions bound by FOS during an epigenetic switch linking inflammation to cancer

**DOI:** 10.1186/1756-8935-8-7

**Published:** 2015-02-14

**Authors:** Joseph D Fleming, Paul G Giresi, Marianne Lindahl-Allen, Elsa B Krall, Jason D Lieb, Kevin Struhl

**Affiliations:** Department of Biological Chemistry and Molecular Pharmacology, Harvard Medical School, Boston, MA 02115 USA; Department of Human Genetics, University of Chicago, 920 E. 58th Street, Chicago, IL 60637 USA

**Keywords:** MCF-10A, Transformation, FAIRE, STAT3, FOS

## Abstract

**Background:**

Transient induction of the Src oncoprotein in a non-transformed breast cell line can initiate an epigenetic switch to a cancer cell via a positive feedback loop that involves activation of the signal transducer and activator of transcription 3 protein (STAT3) and NF-κB transcription factors.

**Results:**

We show that during the transformation process, nucleosome-depleted regions (defined by formaldehyde-assisted isolation of regulatory elements (FAIRE)) are largely unchanged and that both before and during transformation, STAT3 binds almost exclusively to previously open chromatin regions. Roughly, a third of the transformation-inducible genes require STAT3 for the induction. STAT3 and NF-κB appear to drive the regulation of different gene sets during the transformation process. Interestingly, STAT3 directly regulates the expression of NFKB1, which encodes a subunit of NF-κB, and IL6, a cytokine that stimulates STAT3 activity. Lastly, many STAT3 binding sites are also bound by FOS and the expression of several AP-1 factors is altered during transformation in a STAT3-dependent manner, suggesting that STAT3 may cooperate with AP-1 proteins.

**Conclusions:**

These observations uncover additional complexities to the inflammatory feedback loop that are likely to contribute to the epigenetic switch. In addition, gene expression changes during transformation, whether driven by pre-existing or induced transcription factors, occur largely through pre-existing nucleosome-depleted regions.

**Electronic supplementary material:**

The online version of this article (doi:10.1186/1756-8935-8-7) contains supplementary material, which is available to authorized users.

## Background

Oncogenic transformation is often driven by the genetic alteration of protein kinases, which results in inappropriate activity of downstream transcription factors (TFs). These TFs cause changes in gene expression that ultimately mediate the phenotypes of a transformed cancerous cell type such as invasion, metastasis, loss of contact-mediated growth inhibition, uncontrolled proliferation, and tumor formation. The signal transducer and activator of transcription 3 protein (STAT3) is an important mediator of the transcriptional changes during this process, and it is implicated in many types of cancers [1–4].

STAT3 is a DNA-binding transcription factor (TF) that is part of a larger family consisting of seven members. STAT3 contains an SH2 domain, and it is phosphorylated at tyrosine 705 (Tyr^705^) and serine 727 (Ser^727^) in response to many cytokines and growth factors [5]. Tyr^705^ phosphorylation is critical for dimerization, nuclear localization, and gene activation, while Ser^727^ phosphorylation plays a minor role in modulating STAT3 activity. The oncogenic kinase SRC phosphorylates STAT3 *in vitro* and co-immunoprecipitates with STAT3 from cellular extracts [6]. Many primary tumors contain constitutively activated STAT3, and 30%–60% of primary breast cancer specimens contain Tyr^705^-phosphorylated STAT3. Inhibition of STAT3 activity impairs proliferation and induces apoptosis [7]. Conversely, overexpression of an active form of STAT3 (STAT3-C) in immortalized human fibroblasts [8] or the mammary epithelial cell line MCF-10A [9] is sufficient to induce transformation. STAT3 has many biological functions in normal cells and, like other oncogenes, is insufficient on its own to cause cancer. In the case of MCF-10A, overexpression of STAT3-C altered the expression of 199 genes [9], although it was unclear how many of these were due to direct STAT3 binding.

In previous work, we utilized an inducible model of cellular transformation to identify transcriptional regulatory circuits important in oncogenesis [10, 11]. This model involves MCF-10A, a non-transformed mammary epithelial cell line [12] containing ER-Src, a derivative of the Src kinase oncoprotein (v-Src) that is fused to the ligand-binding domain of the estrogen receptor [13]. Treatment of such cells with tamoxifen rapidly induces Src, and morphological transformation is observed within 24–36 h. Unlike the parental cell line, the transformed cells form foci and colonies in soft agar, show increased motility and invasion, form mammospheres, and confer tumor formation in mouse xenografts. This model provides the opportunity to kinetically follow the pathway of cellular transformation in a manner similar to that used to study viral infection and other temporally ordered processes.

In this inducible transformation model, transient activation of Src triggers an inflammatory response that results in an epigenetic switch between non-transformed and transformed cells [10]. This epigenetic switch is mediated by an inflammatory positive feedback loop involving the NF-κB and STAT3 transcription factors, Lin28B, IL6, microRNAs (Let-7, miR-181b, miR-21), and tumor suppressor genes (PTEN and CYLD) [10, 14]. Here, we use this model to further explore the transcriptional regulatory network involved in inflammation-mediated transformation on a genome-wide scale, focusing on STAT3. Specifically, we map open chromatin regions (FAIRE-seq), STAT3 binding sites (ChIP-seq), and STAT3-dependent gene expression during the process of cellular transformation. Our results uncover biological pathways regulated directly by STAT3 and identify new regulatory connections between STAT3 and NF-κB networks. Analysis also suggests an important role for FOS in STAT3-dependent transcription.

## Results and discussion

### Accessible chromatin regions are largely unchanged during cellular transformation

Genome-wide identification of nucleosome-depleted regions by formaldehyde-assisted isolation of regulatory elements (FAIRE)-seq or DNase-seq reveals that human cells typically have approximately 100,000 such open chromatin regions, with many differences in the genomic location of such regions among different cell types [15, 16]. Cell type-specific open chromatin regions tend to be enhancers, and they are significantly enriched for the DNA motifs of TFs pertinent to the establishment/maintenance of that cell type. Such cell type-specific determinant TFs are often “pioneer” factors that, perhaps with cooperating TFs, can access their cognate DNA motif in the context of a nucleosome, whereupon they recruit nucleosome remodeling complexes and histone acetylases that open chromatin structure and permit binding of other factors.

**Figure 1:**
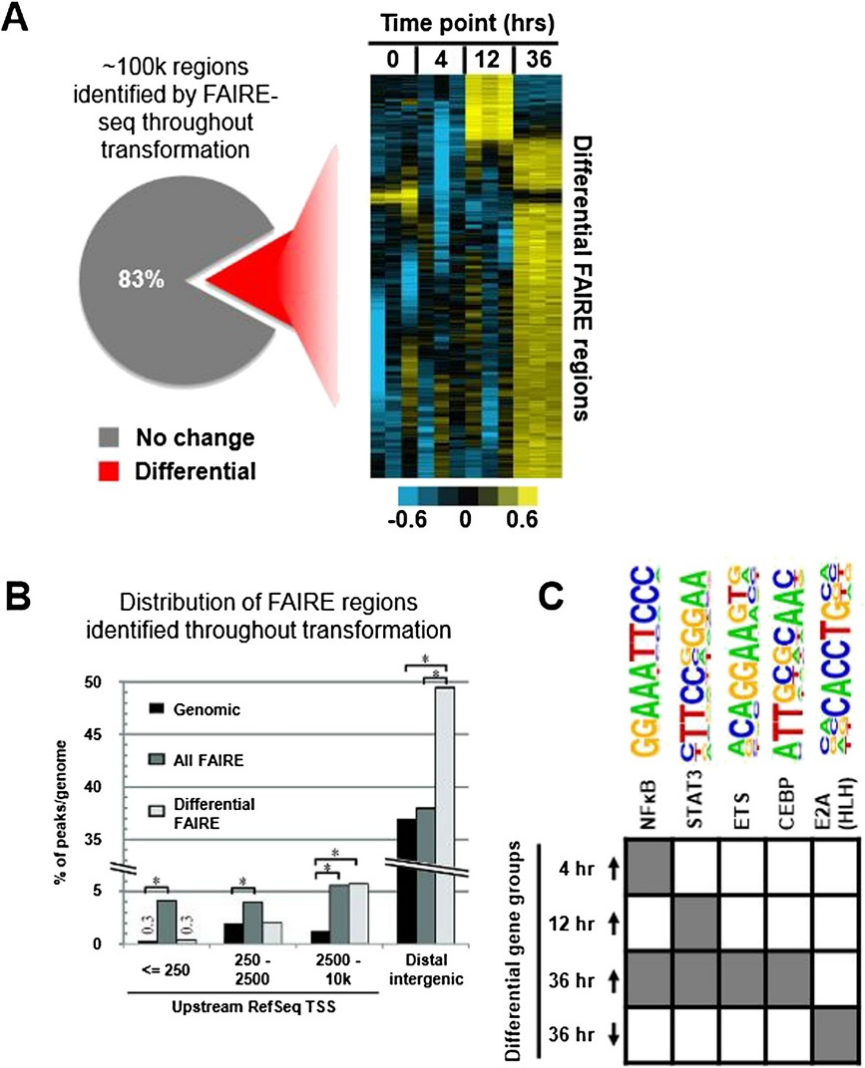
**Open chromatin regions identified by FAIRE before and during transformation. (A)** The pie chart represents the approximately 100,000 non-redundant FAIRE sites identified throughout transformation. A minority (17%) changed during transformation and is shown as the rows of the heatmap on the right. The columns of the heatmap display the replicates for FAIRE-seq at each of the indicated time points. For each FAIRE site, the normalized read count was calculated and the data in the heatmap was median centered by rows (yellow = increased and blue = decreased signal). **(B)** Distribution of FAIRE sites relative to RefSeq gene transcription start site (TSS) annotations. **(C)** A select set of TF motifs are enriched at FAIRE sites associated with genes whose RNA levels are differentially regulated during transformation. HOMER identified TF binding site motifs enriched at FAIRE sites within ±500 kb of the transcription start site of genes differentially expressed at the indicated time points (rows). Gray indicates an enriched motif (*P < 0.005).

We used FAIRE-seq [17, 18] to identify nucleosome-depleted regions in MCF-10A-ER-Src cells that were untreated (0-h control) or treated with tamoxifen (TAM) for 4, 12, or 36 h. Across all samples, we identified ~100,000 non-redundant FAIRE regions (Figure 1A) that correspond well to those observed in other breast cell lines and align with known TF binding sites [19] (Additional file 1: Figures S1 and S2). Coupled to this, we used DNA microarrays to identify genes that were differentially expressed during the transformation time course and identified 116 and 1,483 genes at 4 and 24 h post TAM treatment, respectively. Despite the major phenotypic and transcriptional changes observed during transformation, only ~17% of FAIRE regions exhibit an amplitude change in at least one time point during transformation (Figure 1A). Moreover, the majority of these changes are not the result of *de novo* formation of FAIRE regions, but rather due to an increase in signal at sites that already had low-amplitude FAIRE enrichment (Additional file 1: Figures S3 and S4). Such differential FAIRE regions are observed mainly at the 36-h time point (Figure 1A), yield no significant gene ontology (GO) terms (Methods), and are located mostly at regions greater than 10 kb from the transcription start site (TSS) (Figure 1B).

### Transcription factor binding motifs that are overrepresented in FAIRE sites near genes with transformation-altered expression are generally overrepresented in accessible chromatin regions prior to transformation

Only a small minority of FAIRE regions change during transformation, and therefore, one possibility is that the phenotypic and gene expression changes observed were mediated primarily through chromatin that was already open in the cells prior to transformation. Indeed, TF binding motifs that occurred within open chromatin regions present prior to transformation are also enriched in open chromatin near genes that were induced at particular time points during the transformation process (Figure 1C). Interestingly, at the 4-h time point, NF-κB motifs are overrepresented in open chromatin near induced genes, whereas STAT3 motifs are not (Figure 1C). Conversely, STAT3 motifs, but not those of NF-κB, are overrepresented in open chromatin among genes induced at the 12-h time point. At the 36-h time point, NF-κB, STAT3, ETS, and CEBP motifs are all enriched among induced genes, whereas E2A motifs are enriched among downregulated genes.

### Transformation-dependent binding of STAT3 is restricted to motifs found within FAIRE regions

**Figure 2:**
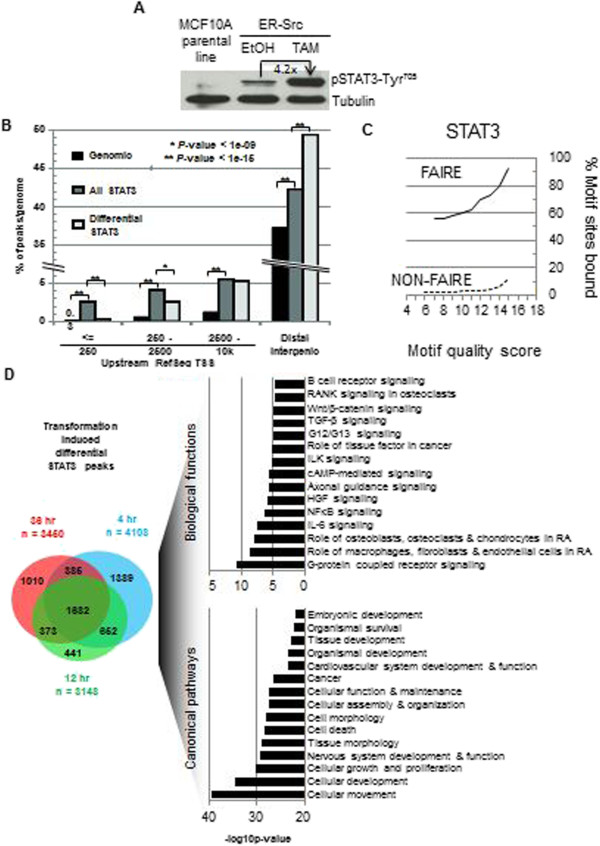
**STAT3 binding profiles before and during transformation. (A)** Western blots of protein extracts from TAM-treated MCF10A-ER-Src cells. **(B)** Distribution of STAT3 occupancy at RefSeq gene features. “All STAT3” represents all treatments/time points, i.e., cumulative. “Differential” refers to STAT3 occupancy in transformed cells only. **(C)** Occupancy of STAT3 DNA binding site motifs by STAT3 protein, as a function of increasing motif quality score, within FAIRE-seq regions and in non-FAIRE regions. Data was from all STAT3 conditions/time points. **(D)** Gene ontology terms associated with transformation differential STAT3 ChIP-seq sites and the overlap between at 4, 12, and 36 h post TAM treatment.

STAT3 is critical for oncogenic transformation, and the STAT3 motif is overrepresented within FAIRE regions associated with differentially regulated genes (Figure 1C, and see below). STAT3 RNA levels increase modestly by ~50% during transformation (Additional file 1: Figure S5), and STAT3 activity, as measured by Tyr^705^ phosphorylation, increases ~4-fold (Figure 2A). Using chromatin immunoprecipitation (ChIP)-seq, we identify 26,783 STAT3 binding sites (*P* value <10^−9^) in non-transformed ER-Src cells, presumably representing a basal level of ER-Src signaling and hence STAT3 function. Upon transformation, STAT3-bound sites (as defined by the same statistical cutoff) more than double at 4 h (77,262), 12 h (67,015), and 36 h (74,584) post induction. In accord with STAT3 binding in macrophages [20], most STAT3 sites are located within introns and regions located away from RefSeq gene features, with only 7% of STAT3 binding sites located less than 2,500 bp upstream of a RefSeq TSS (Figure 2B). Approximately 15,000 genes contain at least one STAT3 binding site within their putative regulatory DNA domain as defined by GREAT (Methods), and only 3% of STAT3 sites are not associated with a putative regulatory DNA domain.All STAT3 binding during the transformation process is restricted almost exclusively to sites within FAIRE regions that existed prior to induction of transformation. Further, FAIRE regions containing the STAT3 motif (Figure 2C) are exceptionally well occupied by STAT3. At a motif quality score of 14 (near-perfect canonical motif), 80% of STAT3 motifs within FAIRE regions are bound by STAT3, in contrast to 8% of motifs with a quality score of 14 outside of FAIRE regions (Figure 2C). Thus, binding of STAT3 to its motif is largely limited to nucleosome-depleted genomic loci.

The vast majority of STAT3 binding sites observed during the transformation process are also observed in non-transformed cells, albeit with a lower signal that falls below our significance cutoff. Only 5% of STAT3 binding sites were deemed significant in transformed cells but not in non-transformed cells and also exhibit a greater than 5-fold increase in binding amplitude by ChIP-seq during transformation. These few strongly induced peaks are preferentially located at sites distal to RefSeq TSSs (Figure 2B and Additional file 1: Figures S6 and S7) and will hereafter be termed “differential STAT3 sites.” Only 0.24% of differential STAT3 sites occur at differential FAIRE regions, consistent with the observation that most STAT3 binding specific to the transformation process occurs at chromatin that was open prior to transformation.

### STAT3 binding and STAT3-dependent gene expression during transformation are associated with an inflammatory signature

Differential STAT3 binding sites are significantly overrepresented among genes linked to inflammation (IL6, NF-κB, and TGF-β signaling; Figure 2D). STAT3 sites are also overrepresented within regulatory regions of genes involved in cellular movement, growth and proliferation, cell death, and embryonic development (Figure 2D), key processes all linked to cancer.

**Figure 3:**
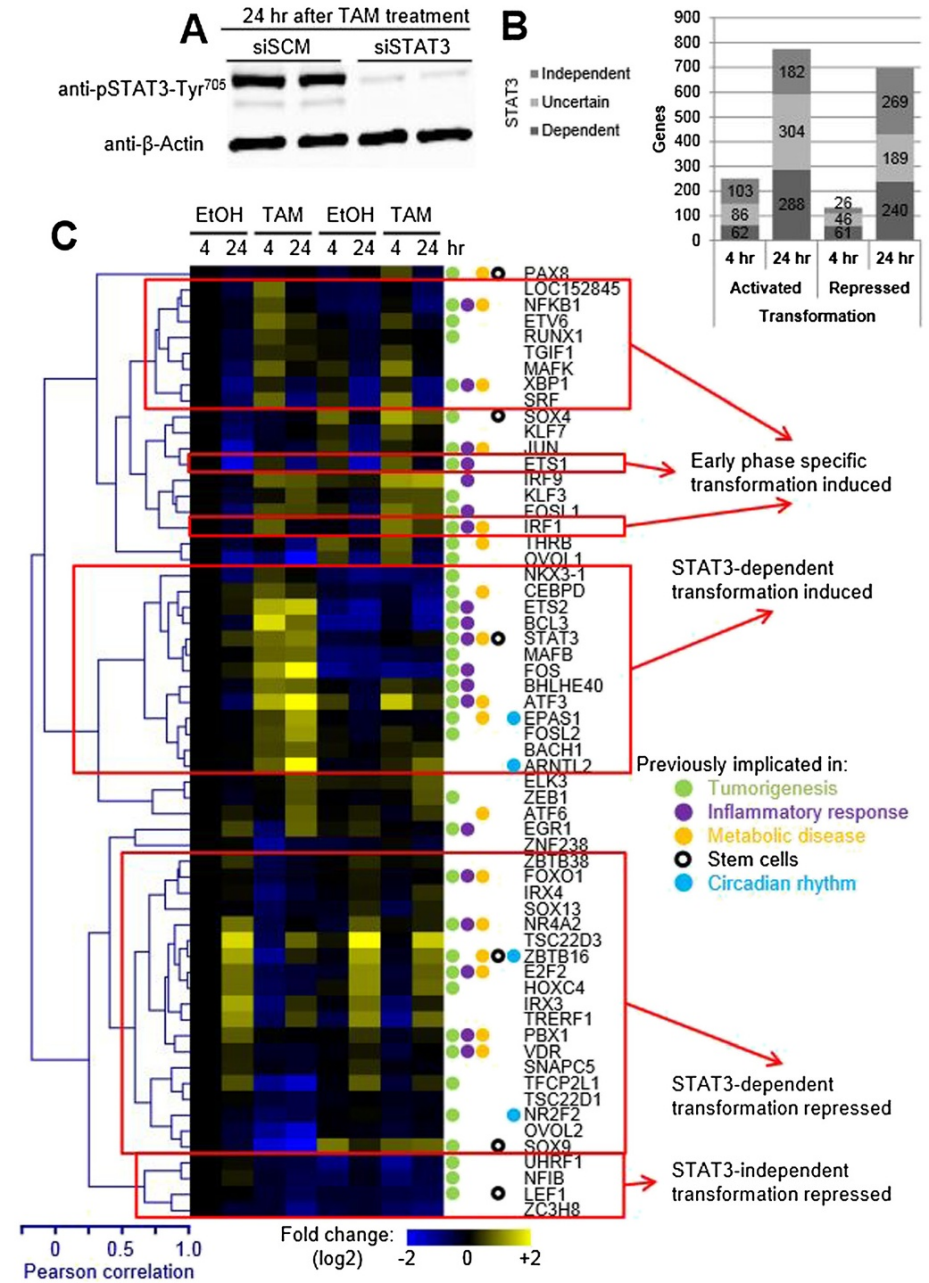
**Differentially expressed transcriptional regulators during transformation. (A)** Western blots of protein extracts from TAM-treated MCF10A-ER-Src cells done in parallel to RNA samples used for expression microarray analysis. STAT3 protein levels are reduced >25-fold upon siSTAT3 knockdown by 24 h. **(B)** The numbers of genes differentially up- or downregulated during transformation at 4 and 24 h post ER-Src activation and their dependence on STAT3. **(C)** Shown are the RNA expression levels at 4 and 24 h post EtOH or TAM treatment in samples transfected with siSCM (scrambled control) or siSTAT3. RNA levels are expressed as fold change over 4-h EtOH- and siSCM-treated samples. All TFs differentially regulated (*P* value <10^−4^, >log_2_ 0.5-fold change) upon ER-Src activation at 4 or 24 h post TAM treatment in siSCM-transfected cells. TFs were clustered, and red boxes indicate TFs whose transcriptional response to treatments is correlated. Those TFs known to be involved in tumorigenesis, inflammatory response, metabolic disease, “stemness,” and the circadian rhythm are indicated by colored circles.

**Figure 4:**
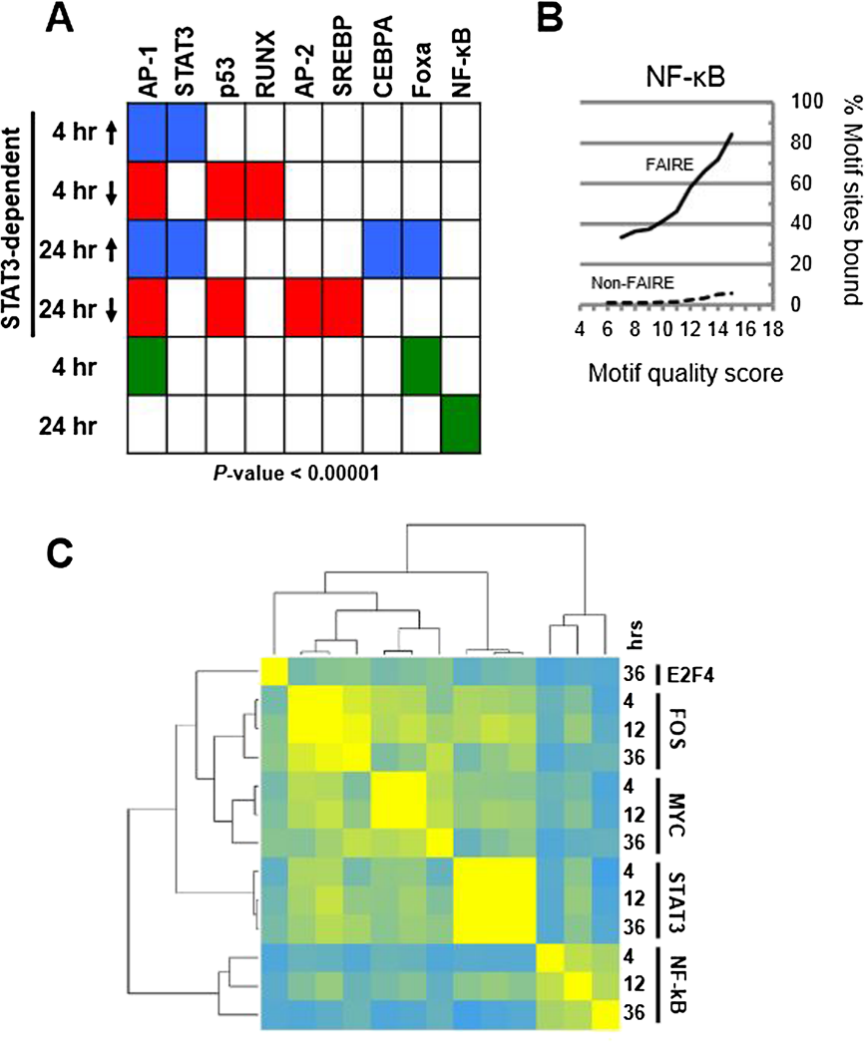
**NF-κB binds a subset of FAIRE sites independent of STAT3. (A)** Transcription factor binding motifs enriched within FAIRE sites that were ±500 kb from the TSS of the genes in each indicated group. STAT3-dependent gene groups are separated based on whether their expression increases or decreases in response to siSTAT3. **(B)** Occupancy of NF-κB DNA binding site motifs, as a function of increasing motif quality score, within FAIRE-seq regions and in non-FAIRE regions. **(C)** Correlation matrix of the ChIP-seq signal at FAIRE sites. Read counts for each of the factors and conditions measured by ChIP-seq, normalized to input, were used to calculate the correlation matrix. The resulting correlation matrix is symmetric (same datasets represented on rows and columns) which was clustered by row and column to order datasets based on the similarity of binding within FAIRE sites. The correlation matrix indicates that NF-κB correlates well with itself but not to other factors, indicating that NF-κB binds an independent subset of sites. FOS and STAT3 however occupy the same FAIRE sites more frequently than other factors.

To identify genes whose expression depends on STAT3 during transformation, we performed microarray analysis in TAM-treated cells in which STAT3 protein (Figure 3A) and RNA levels were reduced more than 10-fold using siRNA (Additional file 1: Figure S5). Approximately one third of genes normally differentially regulated during transformation are dependent on STAT3 (Figure 3B). Genes that show differential upregulation during the transformation correlate both with the number of differential STAT3 sites and with increased STAT3 occupancy during transformation (Additional file 1: Figure S6). These relationships are not observed with genes downregulated during transformation, suggesting that STAT3 directly regulates only genes that are activated during transformation. Interestingly, the p53 motif was found enriched in downregulated genes (Figure 4A), and in this regard, our previous analysis identified p53 as a common node linking inflammatory signals and cancer transformation to metabolic syndrome [11]. The genes encoding p53 and p63 are both upregulated in response to transformation at 4 h, indicating that they are induced early by STAT3, only to be downregulated as transformation progresses (Additional file 1: Figure S8).

Gene ontology analysis of genes differentially regulated during transformation in a STAT3-dependent or STAT3-independent manner reveals that STAT3 is more important for regulating genes involved in the inflammatory response and less important for genes that are involved in cellular metabolism, especially at the 24-h time point (Additional file 1: Figure S9). ETS2, BCL3, FOS, ATF3, ARNTL2, and TSC22D3 are the topmost differentially regulated TFs during transformation (Figure 3C). All of these are STAT3 dependent and linked to tumorigenesis and/or inflammation. Taken together, these observations are consistent with the central role of direct STAT3 targets in transformation.

### STAT3 and NF-κB drive alternate and cooperative pathways during transformation

It is well known that STAT3 and NF-κB are important TFs involved in inflammation and cancer [21, 22], and STAT3 has a major role in activating inflammatory genes during the transformation process in our inducible model. Ingenuity Pathway Analysis (IPA) of genes regulated during transformation in a STAT3-dependent manner at the 4-h time point reveals NF-κB as the central cooperating effector of the perturbed signaling pathways (Additional file 1: Figure S10A). At the later 24-h time point of STAT3-dependent transcription regulation, IPA indicates a switch from NF-κB to FOS acting cooperatively with STAT3, with STAT3 and FOS controlling genes involved in cellular assembly and organization, and development (Additional file 1: Figure S10B). Interestingly, NF-κB was also found to be a central effector molecule at the early and late time points of STAT3-independent transformation-regulated genes (Additional file 1: Figure S10C, D). Specifically, NF-κB could be transcriptionally driving gene networks of carbohydrate metabolism, drug metabolism, and small molecule biochemistry independently of STAT3, thus showing that NF-κB and STAT3 have independent and cooperative roles during transformation.

The STAT3 motif is significantly enriched in the FAIRE regions near STAT3-dependent activated genes, but not STAT3-dependent repressed genes (Figure 4A). STAT3 indirectly regulates ~35% of genes that are repressed during transformation, and yet the only motifs enriched are FOXA and AP-1 at 4 h and NF-κB at 24 h (Figure 4A). As observed for STAT3 (Figure 2C), NF-κB motifs are much more likely to be occupied when they occur within FAIRE sites as compared to the rest of the genome (Figure 4B). A correlation matrix shows that NF-κB is present at a unique subset of FAIRE sites, whereas STAT3 occupancy is more highly correlated with the occupancy of other factors including FOS and MYC (Figure 4C). This suggests that NF-κB regulates a subset of genes that are not directly dependent on STAT3. Network analysis suggests that these genes may be more involved in the metabolic aspects of transformation (Additional file 1: Figure S10).

### GILZ and other TFs are indirectly inhibited by STAT3 during transformation

There is a class of TFs whose expression is repressed early and/or late during transformation in a STAT3-dependent manner (Figure 3C). Many of these TFs also show increased expression 24 h post ethanol (EtOH) treatment (control non-transformed cells that are becoming confluent) in a STAT3-independent manner (Figure 3C). Such STAT3-dependent transcriptional inhibition is likely to be due to direct binding of STAT3 to the target genes. However, STAT3 is not thought of as a transcriptional repressor. GILZ (also known as TSC22D3) is the most dynamically regulated TF of this group, and near its promoter region, an increase in STAT3 occupancy is observed upon TAM treatment compared to an EtOH control (Additional file 1: Figure S11). GILZ is interesting because it inhibits the Ras/Raf signaling pathways, directly interacts with and inhibits NF-κB, and has anti-inflammatory properties [23, 24]. Activated Ras and NF-κB can initiate the inflammatory feedback loop that underlies the epigenetic switch to the transformed state [10]. Thus, it is tempting to speculate that GILZ-mediated inhibition of the inflammatory pathway helps keep cells in the non-transformed state and that STAT3-dependent inhibition of GILZ expression increases inflammation, thereby leading to cellular transformation. Presumably, during the transformation process, STAT3 activates and/or recruits a repressor that directly inhibits GILZ transcription in response to an inflammatory signal. As such, these observations suggest the possibility of a biphasic switch in which the inflammatory molecules (NF-κB, STAT3, Ras/Raf, IL6) are responsible for inhibiting GILZ, whereas GILZ inhibits the inflammatory pathway. Such a biphasic switch could reinforce either the non-transformed or the transformed state at a given point in time.

### STAT3 may cooperate with FOS at target sites during transformation

**Figure 5:**
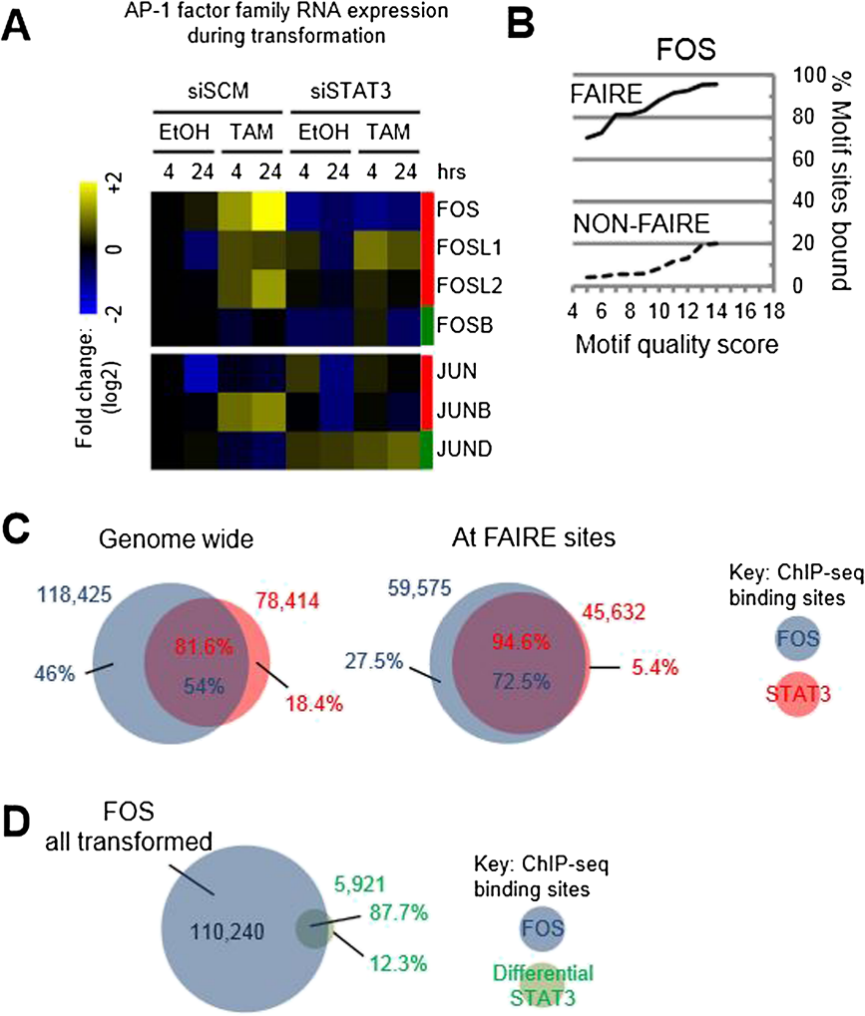
**Cooperation of STAT3 and FOS sites during transformation. (A)** AP-1 factors during transformation and their transcriptional dependence on STAT3. Shown are the normalized RNA expression microarray levels at 4 and 24 h post EtOH or TAM treatment in samples transfected with siSCM (scrambled control) or siSTAT3. **(B)** Occupancy of FOS DNA binding site motifs, as a function of increasing motif quality score, within FAIRE-seq regions and in non-FAIRE regions. **(C)** All FOS and STAT3 sites from each time point that directly overlap or overlap only at FAIRE sites. **(D)** Transformation-dependent differential STAT3 sites directly overlapping all FOS sites from 4, 12, and 24 h post induction.

The bias of differential STAT3 sites towards distal intergenic regions is likely to reflect the presence of a cooperating factor(s). The enrichment of AP-1 motifs within FAIRE regions (Additional file 1: Figure S1) and the identification of FOS (a component of AP-1) as a cancer signature gene linking inflammation with metabolic syndrome in ER-Src and in a fibroblastic cell line [11] suggest the importance of AP-1 factors. AP-1 is composed of FOS (FOS, FOSL1, FOSL2, FOSB) and JUN family (JUN, JUNB, JUND) members, some of which are significantly differentially expressed during transformation (Figures 3C and 5A). FOS itself is one of the most differentially expressed STAT3-dependent TFs during transformation, and STAT3 sites are present in the FOS-proximal promoter region (not shown). In addition, in a STAT3-dependent manner, FOSL2 and JUNB are activated during transformation, whereas JUND is repressed (Figure 5A and Additional file 1: Figure S12).These observations prompted us to explore FOS binding genome-wide during transformation. FOS appears to be less dependent on its canonical motif for binding than STAT3 or NF-κB but shows an equally marked preference for open chromatin (FAIRE regions) (Figure 5B). Importantly, the increase in STAT3 occupancy across the genome during transformation is closely associated with FOS binding (Figure 5C, D). Of all STAT3 sites, 82% directly overlap a FOS site, with a higher overlap within FAIRE sites (95%), which persists throughout transformation. Nearly all (88%) differential STAT3 sites are directly associated with a FOS site (Figure 5D), with only 25% associating with a differential FOS site. The regulatory regions bound by FOS at genes differentially regulated during transformation highlight its importance to many STAT3-regulated processes such as G protein-coupled receptor signaling, NF-κB signaling, cellular movement, and cell death (not shown). These observations suggest that FOS may cooperate with STAT3 in many key processes of transformation.

### Additional molecular connections in the transcriptional regulatory circuit that underlies the epigenetic switch

**Figure 6:**
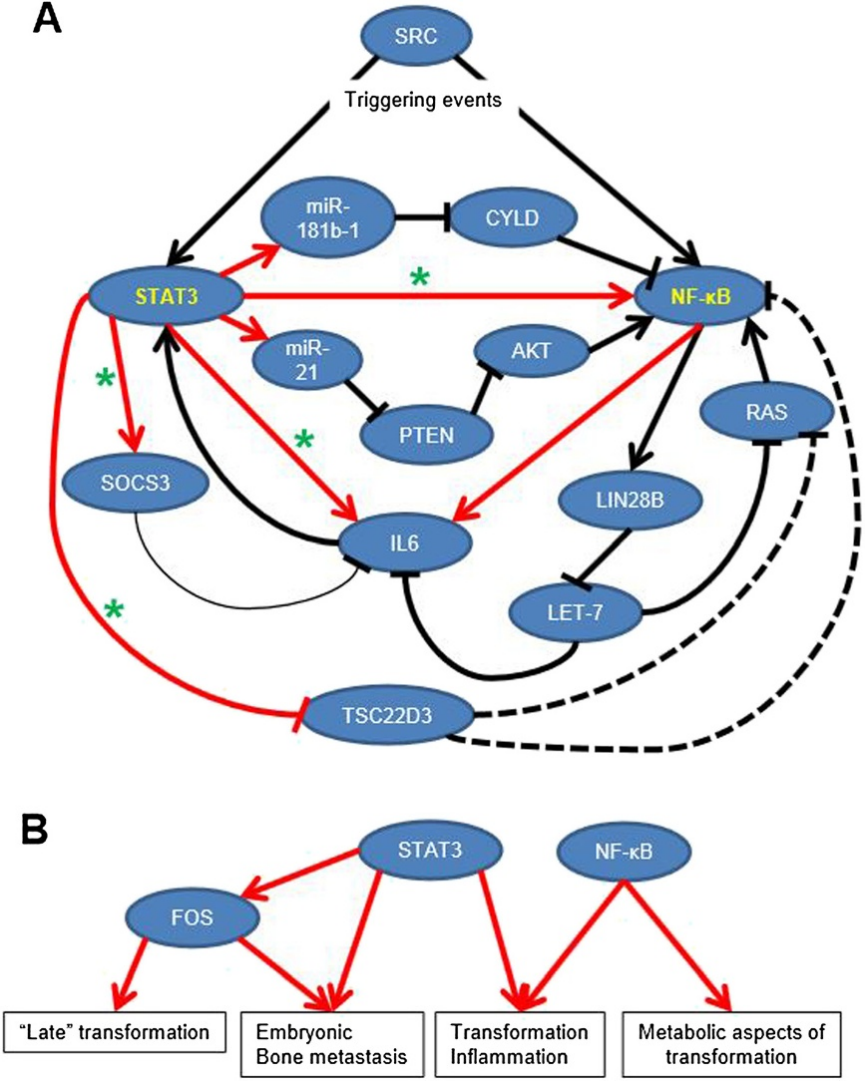
**A refined model for an epigenetic switch that initiates and maintains transformation. (A)** Model of the epigenetic switch and its positive feedback loops that mediate transformation. Dashed lines indicate predicted interactions based on literature findings. Red lines indicate direct transcriptional regulation. Black lines ending in an arrow or perpendicular slash indicate known positive and inhibitory interactions, respectively, within MCF10-ER-Src based on previous experimental findings and this work. A thin line indicates weak activity. A green star represents interactions based on data presented in this work. **(B)** The major phenotypic effects and processes mediated by STAT3, NF-κB, and FOS.

The current view of the transcriptional circuit that mediates an epigenetic switch to the transformed state (Figure 6A, B) is that NF-κB is activated rapidly upon induction, whereupon it directly and indirectly (via Lin28B and Let-7 miRNA) upregulates IL6 expression leading to STAT3 activation [10]. STAT3 is also part of this switch, by directly activating transcription of miR-21 and miR-181b, which activate NF-κB via inhibiting the tumor suppressor genes PTEN and CYLD [14]. Interestingly, we show that NFKB1 (which encodes the p105/p50 subunit of NF-κB) is also a direct transcriptional target of STAT3. Its RNA levels increase early during transformation in a STAT3-dependent manner (Figure 3C), and differential STAT3 binding sites are found within an intron of NFKB1 and just downstream of the gene, with additional non-differential sites located upstream (not shown). In addition, we show that IL6 not only stimulates STAT3 function but is a direct STAT3 target gene, as STAT3 occupies its promoter and transcriptional induction is STAT3 dependent (not shown, Additional file 1: Figure S5). Hence, there is additional complexity to the inflammatory feedback loop that involves STAT3 transcriptionally upregulating NF-κB and IL6, both of which activate STAT3. This aspect of the epigenetic switch is probably more relevant during the later stages of transformation because it is dependent on new protein production. It is unclear whether an increase in *STAT3* mRNA is important for transformation, but it is likely to contribute to the transformed state and the maintenance of STAT3 protein levels.

## Conclusions

Gene expression changes caused by induction of the Src oncoprotein during the transition from a non-transformed to a transformed breast cell line, whether driven by pre-existing or induced transcription factors, occur largely through pre-existing nucleosome-depleted regions. Motifs overrepresented in FAIRE sites near genes that change during transformation are also overrepresented in chromatin regions that were accessible prior to transformation. During the transformation process, STAT3 and NF-κB appear to drive alternate and cooperative pathways, GILZ and other TFs are indirectly inhibited by STAT3, STAT3 appears to cooperate with FOS at target sites, and NFKB1 (which encodes the p105/p50 subunit of NF-κB) is a direct transcriptional target of STAT3. These additional complexities to the inflammatory feedback loop are likely to contribute to the epigenetic switch.

## Methods

### Tissue culture

MCF10A-ER-Src cells were grown and ChIP DNA was isolated as per standard ENCODE protocols. A detailed protocol is available at http://genome.ucsc.edu/ENCODE/. Cultures were grown to 70% confluency; then treated with either EtOH or TAM for 4, 12, 24, or 36 h, as detailed in [11]; and harvested for DNA, protein, or RNA as detailed below.

### FAIRE-seq

Cells were grown as above and a full detailed FAIRE-seq protocol is available at http://genome.ucsc.edu/ENCODE/ and [17]. Briefly, cells were fixed by adding 37% formaldehyde directly to growth media to a final concentration of 1% and incubated at room temperature on an orbital shaker at 80 rpm for 5 min. Crosslinking was quenched by adding 2.5 M glycine to a final concentration of 125 mM and incubated for 5 min at room temperature while continuing to shake. Media containing formaldehyde was removed and ice-cold PBS was added to cover the cell layer. Cells were scraped from the plate and washed two more times with ice-cold PBS to ensure all residual media was removed.

Cells were resuspended in 1 ml of lysis buffer (2% Triton X-100, 1% SDS, 100 mM NaCl, 10 mM Tris-Cl, pH 8.0, 1 mM EDTA) per 10^7^ cells and transferred to a 2-ml screw-capped tube with rubber seal containing 1 ml of 500 μM glass beads. Cells were disrupted using a mini bead-beater (Mini-BeadBeater-8, BioSpec Inc.) set to homogenize for five 1-min sessions with 2-min incubations on ice between sessions. Lysate was transferred to 1.5-ml tubes in 300-μl aliquots and sonicated for 15 min using a Bioruptor UCD-200 (Diagenode) set to pulse on high for 30 s followed by 30 s of rest in a 4°C water bath. Cellular debris was cleared from the lysate by spinning at 15,000 × *g* for 5 min at 4°C.

An equal volume of phenol/chloroform (Sigma #P3803 phenol, chloroform, and isoamyl alcohol (25:24:1) saturated with 10 mM Tris, pH 8.0, 1 mM EDTA) was added to the lysate, vortexed, and spun at 12,000 × *g* for 5 min, and the aqueous fraction was transferred to a fresh 1.5-ml tube. An additional phenol/chloroform extraction was performed by adding an equal volume of phenol/chloroform to the isolated aqueous fraction. Finally, an equal volume of chloroform (Fluka BioChemika 25666, chloroform and isoamyl alcohol (24:1)) was added to the aqueous fraction and spun at 12,000 × *g* for 5 min.

DNA recovered in aqueous phase was precipitated by adding 3 M sodium acetate (pH 5.2) to a final concentration of 0.3 M and adding 1 μl of 20 mg/ml glycogen, which were mixed by inverting. Two volumes of 95% ethanol were added, mixed by inverting, and incubated at −20°C overnight. DNA was precipitated by spinning at 15,000 × *g* for 30 min at 4°C, and the DNA pellet was washed with 500 μl ice-cold 70% ethanol. Upon removing the supernatant, the pellet was dried in a speed-vac. The FAIRE DNA was resuspended in 50 μl of 10 mM Tris-HCl, pH 7.5, and incubated with 1 μl of 10 mg/ml RNase A for 1 h at 37°C. The FAIRE DNA was cleaned up by performing an additional phenol/chloroform reaction and ethanol precipitation (as described above).

Libraries were prepared using 100 ng of FAIRE DNA. The ends of the DNA fragments were made blunt using the End-It™ DNA End-Repair Kit (Epicentre #ER0720), an A-overhang was added using Klenow exo- (Epicentre #KL06041K), and double-stranded adapters containing a T-overhang (Illumina #1000521) were ligated to the DNA fragments using T4 DNA ligase (NEB #M0202S). Libraries were then amplified using PfuUltra II (Stratagene #600670) and primers complimentary to the adapters (Illumina #1000537 and 1000538). Amplified products were loaded into a 2% agarose gel using sample loading buffer (50 mM Tris, pH 8.0, 40 mM EDTA, 40% *w*/*v* sucrose) and run at 120 V for 1 h. The brightest portion of the smear was excised (150–200 bp), and DNA was recovered using the Qiagen Gel Extraction Kit (#28704). FAIRE DNA libraries were sequenced using the Illumina GAII system to generate 36-bp single-end reads.

### Analysis of FAIRE-seq data

The raw reads for each FAIRE-seq sample (three replicates at each time point) were aligned to the UCSC hg19 build of the human genome using *Bowtie*[25]. Each aligned position was allowed to occur up to four times throughout the genome, where one was selected at random for each of those positions that were not unique. The set of enriched regions were then identified by *ZINBA*[26], using 300-bp windows with 75-bp offsets [see Additional files 2 and 3]. The background and enriched components were modeled using G/C content and an interaction term between mapability and local background estimate. No peak refinement was included in this analysis. Overlap between datasets and with genomic features were carried out using *BEDTools*[27]. Data has been submitted to the NCBI SRA database under project PRJNA270300.

The union set of FAIRE sites enriched throughout the time course were combined and analyzed to identify sites with either an increase or a decrease of FAIRE signal. The occurrence of each active regulatory element was evaluated to determine the extent to which it differed with respect to the other time points, either present at one time point and absent at the others or vice versa. The count of reads aligning to each region identified in the union set was recorded for each time point and normalized by the total number of sequencing reads for that sample. Here, the mean normalized values for the replicates for each time point were compared to the mean values of the replicates for the remaining time points and divided by the sum of standard deviations. To estimate the differences expected by chance, the individual replicates were randomly permuted and the scores were calculated as above. Those FAIRE sites whose scores exceeded the scores calculated at random (0.05) were called as differential. Thus, a small fraction of called differential sites are likely to be false positives.

### Motif analysis of FAIRE sites

Motif enrichment for the set of sequences within all FAIRE sites discovered throughout the time course and those identified as differential was performed using *HOMER*[28]. For all FAIRE sites, motif enrichment was calculated using the sequence at ±5 kb flanking each FAIRE site while the differential FAIRE sites were compared to the set of static FAIRE sites.

Motif enrichment for differentially expressed genes was calculated by identifying the set of FAIRE sites within ±500 kb of the genes in each group. The sequence within these sites was compared to the set of FAIRE sites identified from nearby randomly selected gene groups using HOMER (*P* < 0.005). These randomly selected gene groups contained the same number of genes.

### ChIP-seq and peak calling

ChIP DNA (two biological replicates) was prepared as above (a detailed protocol is available at http://genome.ucsc.edu/ENCODE/) and was immunoprecipitated with anti-phospho-STAT3 antibody (Cell Signaling, 9131) and anti-FOS antibody (Santa Cruz, SC-7202x), or input DNA (three biological replicates) were end repaired with calf intestinal alkaline phosphatase (New England Biolabs, USA) and sent for sequencing to the Stanford Center for Genomics and Personalized Medicine. ChIPs for anti-NF-κB (p65, SC-109) anti-E2F4 (SC-866x), and anti-MYC (SC-764) were prepared as above but using one biological replicate. Library preparation and Illumina (USA) sequencing were carried out as per Illumina protocols, and a detailed protocol is available at http://genome.ucsc.edu/ENCODE/. Sequence reads (32 nucleotides) were mapped to the *Homo sapiens* genome (hg19) using *Bowtie*[25], allowing ≤2 mismatches per read, and reads with >10 reportable alignments were discarded. Binding sites were called using *MACS* v1.4 [29] at a *P* value threshold of 10^−9^, “auto” redundant read setting, using input to control for local genomic biases. *PeakSplitter*[30] was used to split *MACS* called peaks into subpeaks of local maxima using default settings. STAT3 subpeaks were called as differential if they did not overlap a STAT3-bound subpeak in the non-transformed control population (peaks called at 10^−9^*P* value) and had a fold change greater than the mean + 1× standard deviation of all peaks within the population. Fold change in STAT3 ChIP-seq signal was calculated as read counts per region per million mapped reads divided by the corresponding control ChIP signal of that region. A smoothing value of 10 was added to the read count of each region in the transformed and control samples. Data has been submitted to the NCBI SRA database under project PRJNA270300 [see Additional files 4, 5, 6, 7, 8, 9, 10, 11, 12, and 13].

### Annotation of STAT3 sites to RefSeq TSSs

STAT3 peak summits, defined as the local maxima of read counts within a peak, were mapped to the nearest RefSeq TSS, incorporating strandedness, using an in-house script. Histograms were plotted using the *R* package *ggplot2* using 500-bp bins within a region of ±10 kbp about the TSS centered at 0 bp. The frequency of sites is represented as the Gaussian smoothed kernel density estimate with a bandwidth of the standard deviation of the smoothing kernel, calculated using the density function in *R*. The percentage of STAT3 peaks in the proximal upstream region of RefSeq TSSs and located in distal intergenic regions (defined as not within the following RefSeq genic features: −10 kbp upstream of a TSS, +10 kbp downstream of a transcriptional termination site (TTS), intronic, exonic, 5′ UTR or 3′ UTR) was calculated and compared to the percentage of the genome within each category. Significance was calculated using the single-sided binomial test as implemented in the *binom* function in *R*.

### Motif discovery

The top 10,000 (as ranked by *P* value) STAT3 or FOS ChIP-seq subpeak summit locations were determined and the sequence ±50 bp was extracted and repeat masked. A 5-order Markov model was used as the background set and was extracted from the repeat-masked, non-redundant set of FAIRE-seq *cis*-regulatory elements. Parallel *MEME* was run with the following settings: zoops, revcomp, minw = (4–26), and maxw = (6–30). For STAT3 and FOS, the top motif corresponded to the respective known canonical motif. The motif position-specific frequency matrices derived from *MEME* were used as input to *FIMO*[31], and motifs were discovered genome wide at a significance *P* value threshold of 10^–4^.

### Annotation of ChIP-seq and FAIRE-seq regions to gene features and GO analysis (*GREAT*/*IPA*)

Genomic locations of subpeak summits were submitted to the annotation tool *GREAT*[32] using the following parameters: whole genome background set, basal plus extension, proximal upstream = 5 kbp, proximal downstream = 1 kbp, distal = 1 mbp; or whole genome background set, basal, proximal upstream = 5 kbp, proximal downstream = 1 kbp. Therefore, the putative transcriptional regulatory domain of a gene was defined as follows: “Each gene is assigned a basal regulatory domain of a minimum (proximal) distance upstream and downstream of the TSS (regardless of other nearby genes). The gene regulatory domain is extended in both directions to the nearest gene’s basal domain but no more than the (distal) maximum extension (1 Mbp) in one direction.” For *IPA* (Ingenuity Pathway Analysis, Ingenuity Systems, USA; http://www.ingenuity.com), gene probe IDs, with the corresponding log_2_ fold change, were uploaded into and analyzed by *IPA* (build: 140500, content version: 12710793) using default settings. Molecular signaling pathways were visualized using *IPA* where a gray-shaded node represented a subpeak binding site located within the putative regulatory region, as defined by *GREAT*, of that gene/molecule. The biological relationship between two molecules is represented as a line and is based on professionally curated literature findings. The relationships can be direct or indirect.

### siRNA transfections

MCF10A-ER-Src cells were reverse transfected with Dharmacon siRNAs (Thermo Scientific, USA) according to the manufacturer’s protocol. Briefly, cells were seeded in six-well plates containing 25 nM ON-TARGETplus SMARTpool STAT3 or ON-TARGETplus Non-targeting Pool and after 48 h of growth were treated with EtOH or tamoxifen (1 μM) for 4 or 24 h as per [11]. For Western blots, cells were lysed after 72 h (24 h post EtOH/TAM treatment). For expression profiling, RNA was harvested after 4 or 24 h post treatment.

### Gene expression microarrays

RNA (from three biological replicates) was prepared for arrays using the 3′ IVT Express Kit (Affymetrix, USA) as per manufacturer protocol—100 ng RNA and 15 amplification cycles. Amplified RNA was given to the Boston Children’s Hospital microarray core facility for hybridization to GeneChip Human Genome U133 plus 2.0 gene expression arrays (Affymetrix, USA) for hybridization and imaging as per manufacturer protocols. Data has been submitted to the NCBI GEO database under accession GSE64536.

### Western blots

Membranes were probed using mouse-derived anti-phospho-STAT3 antibody from Cell Signaling Technologies, USA, and anti-β-actin from Sigma, USA. Bands were detected with IRDye 800-labeled goat anti-mouse IgG (LI-COR Biosciences, USA) and imaged using an Odyssey infrared imaging system (LI-COR Biosciences, USA).

### Determination of differential gene expression

Gene expression arrays were background corrected and normalized and probe set expression values determined by the mas5 algorithm [see Additional files 14, 15, 16, 17, and 18]. Probe sets were annotated to RefSeq gene IDs using *GREAT*[32] or *DAVID*[33]. Genes determined to be transformation regulated/differential were derived from siSCM-treated samples comparing EtOH to TAM treatments with a *P* value <10^−4^ and with an absolute log_2_ fold change >0.5. Those genes determined to be STAT3 and transformation regulated were determined by comparing EtOH to TAM samples under both siSCM and siSTAT3 conditions. Genes were selected as STAT3 independent if their differential expression was statistically insignificant upon siSTAT3 and had an absolute log_2_ fold change of <0.5 upon siSTAT3. The number of STAT3-dependent and STAT3-independent genes does not equal the total number of genes considered differential by transformation as many genes could not be unambiguously defined as “dependent” or “independent”.

### Differentially regulated TFs

All probe set IDs that were differentially regulated during transformation (see above) were submitted to *DAVID*, and probe sets annotated to the term “transcription factor activity” (GO:0003700) were selected. Normalized expression values for each gene are expressed as log_2_ fold change over siSCM 4-h EtOH-treated samples. Hierarchical clustering of the resultant expression matrix was carried out using the Pearson correlation and average linkage using the software package *TMEV*[34].

### Annotation of STAT3 sites to differentially expressed genes

Differentially expressed genes were defined as above. Non-redundant probe sets were used, discarding the probe set with the highest *P* value. “Promoter” regions are defined as −2,500 to +500 bp from RefSeq TSS. “Upstream/downstream” regions are defined as ±50 kbp from the RefSeq TSS, excluding the promoter region. The number of transformation-specific differential STAT3 ChIP-seq peaks were counted within these regions, normalized to peaks per 1 kbp, and plotted using a 1,000 gene rolling mean.

## Electronic supplementary material

Additional file 1: **Supplementary Figures S1 to S12.** Supplementary supporting evidence. For a description of each figure, see its associated legend. (PDF 8 MB)

Additional file 2: **All FAIRE sites.** Genomic locations of all FAIRE-seq sites discovered in transformed and non-transformed MCF10A-ER-Src cells. (XLS 2 MB)

Additional file 3: **Differential FAIRE sites.** Genomic locations of all FAIRE sites discovered in MCF10A-ER-Src cells that were determined to be differential during the time course of transformation. (XLS 481 KB)

Additional file 4: **MCF10A-ER-Src STAT3 ChIP-Seq 4-h TAM.** Genomic locations of all STAT3 genomic binding sites, at a significance *P* value ≤1e − 09, discovered by ChIP-Seq in MCF10A-ER-Src cells treated with tamoxifen for 4 h. (XLS 3 MB)

Additional file 5: **MCF10A-ER-Src STAT3 ChIP-Seq 12-h TAM.** Genomic locations of all STAT3 genomic binding sites, at a significance *P* value ≤1e − 09, discovered by ChIP-Seq in MCF10A-ER-Src cells treated with tamoxifen for 12 h. (XLS 2 MB)

Additional file 6: **MCF10A-ER-Src STAT3 ChIP-Seq 36-h TAM.** Genomic locations of all STAT3 genomic binding sites, at a significance *P* value ≤1e − 09, discovered by ChIP-Seq in MCF10A-ER-Src cells treated with tamoxifen for 36 h. (XLS 3 MB)

Additional file 7: **ER-Src STAT3 peaks differential.** Genomic locations of all STAT3 genomic binding sites determined to be differential during the time course of tamoxifen treatment. (XLS 147 KB)

Additional file 8: **MCF10A-ER-Src STAT3 ChIP-Seq EtOH.** Genomic locations of all STAT3 genomic binding sites, at a significance *P* value ≤1e − 09, discovered by ChIP-Seq in MCF10A-ER-Src cells treated with EtOH. (XLS 990 KB)

Additional file 9: **MCF10A-ER-Src FOS ChIP-Seq 4-h TAM.** Genomic locations of all FOS genomic binding sites, at a significance *P* value ≤1e − 09, discovered by ChIP-Seq in MCF10A-ER-Src cells treated with tamoxifen for 4 h. (XLS 6 MB)

Additional file 10: **MCF10A-ER-Src FOS ChIP-Seq 12-h TAM.** Genomic locations of all FOS genomic binding sites, at a significance *P* value ≤1e − 09, discovered by ChIP-Seq in MCF10A-ER-Src cells treated with tamoxifen for 12 h. (XLS 6 MB)

Additional file 11: **MCF10A-ER-Src FOS ChIP-Seq 36-h TAM.** Genomic locations of all FOS genomic binding sites, at a significance *P* value ≤1e − 09, discovered by ChIP-Seq in MCF10A-ER-Src cells treated with tamoxifen for 36 h. (XLS 5 MB)

Additional file 12: **ER-Src FOS peaks differential.** Genomic locations of all FOS genomic binding sites determined to be differential during the time course of tamoxifen treatment. (XLS 129 KB)

Additional file 13: **MCF10A-ER-Src FOS ChIP-Seq EtOH.** Genomic locations of all FOS genomic binding sites, at a significance *P* value ≤1e − 09, discovered by ChIP-Seq in MCF10A-ER-Src cells treated with EtOH. (XLS 5 MB)

Additional file 14: **Differential gene expression at 4-h TAM.** Genes determined to be differentially regulated between MCF10A-ER-Src cells treated with ethanol or tamoxifen for 4 h and with a control scrambled siRNA. (XLS 75 KB)

Additional file 15: **Differential gene expression at 24-h TAM.** Genes determined to be differentially regulated between MCF10A-ER-Src cells treated with ethanol or tamoxifen for 24 h and with a control scrambled siRNA. (XLS 263 KB)

Additional file 16: **STAT3-dependent differential gene expression at 4 h.** Genes determined to be differentially regulated between MCF10A-ER-Src cells treated with ethanol or tamoxifen for 4 h and with a STAT3-specific siRNA. (XLS 27 KB)

Additional file 17: **STAT3-dependent differential gene expression at 24 h.** Genes determined to be differentially regulated between MCF10A-ER-Src cells treated with ethanol or tamoxifen for 24 h and with a STAT3-specific siRNA. (XLS 120 KB)

Additional file 18: **RNA expression values.** Raw RNA MAS5 expression values for MCF10A-ER-Src cells treated with tamoxifen or ethanol, and control siNEG (siSCM) or siSTAT3 at various time points. (XLS 19 MB)

## Abbreviations

ChIP: Chromatin immunoprecipitation
EtOH: Ethanol
FAIRE: Formaldehyde-assisted isolation of regulatory elements
GO: Gene ontology
STAT3: Signal transducer and activator of transcription 3 protein
TAM: Tamoxifen
TF: Transcription factor
TSS: Transcriptional start site.
