# Supplementary material for: STAT3 acts through pre-existing nucleosome-depleted regions bound by FOS during an epigenetic switch linking inflammation to cancer
Source: Epigenetics Chromatin. 2015 Feb 14;8:7. doi: 10.1186/1756-8935-8-7 (PMC4362815; doi:10.1186/1756-8935-8-7)
Supplement: Supplementary file 1 — Additional file 1: Supplementary Figures S1 to S12. Supplementary supporting evidence. For a description of each figure, see its associated legend. (PDF 8 MB) [file 13072_2014_351_MOESM1_ESM.pdf]

S1

**Name**	**log *P*-value**	**Motif**
AP-1	$-1.445e^{4}$	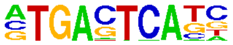
ETS	$-9.829e^{2}$	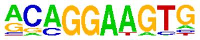
STAT3	$-7.522e^{2}$	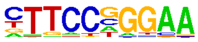
p53	$-7.148e^{2}$	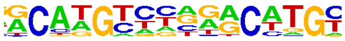
E-box (HLH)	$-6.405e^{2}$	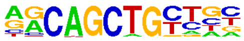
MYC	$-3.090e^{2}$	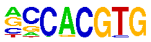
Forkhead	$-3.084e^{2}$	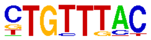
NFκB	$-2.263e^{2}$	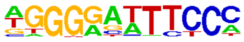

Supplementary Figure S1. The most significant set of transcription factor motifs enriched at all ~100k FAIRE sites identified throughout the transformation. Motif enrichment was calculated using HOMER, with the sequence within all FAIRE sites compared to a background sequence set comprising regions +/- 5kb flanking each FAIRE site.

S2

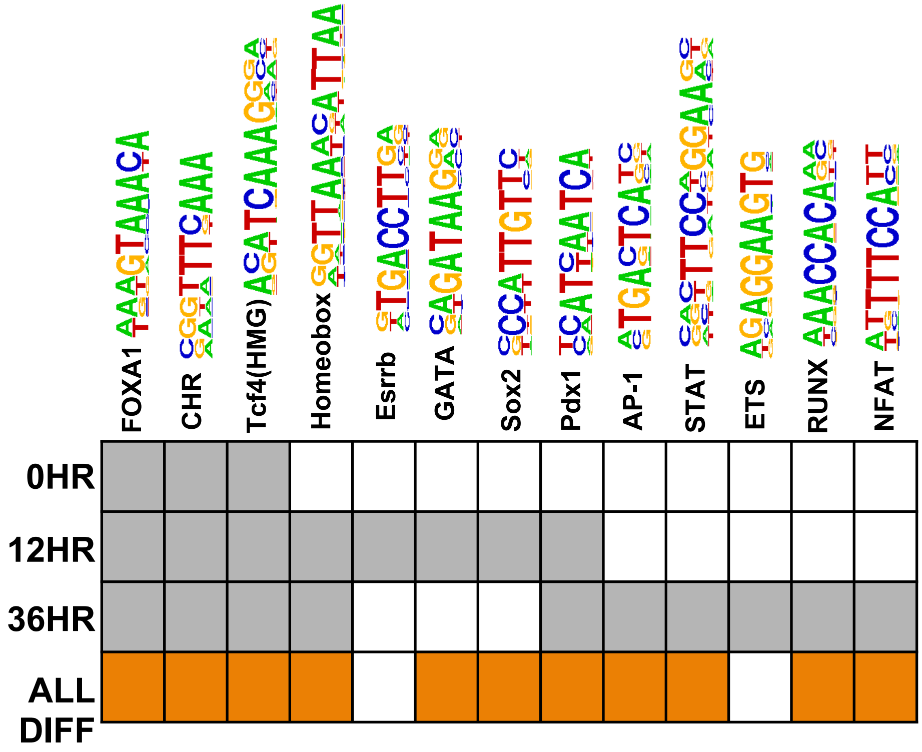

Supplementary Figure S2. Transcription factor binding motifs enriched at differential FAIRE sites. The set of FAIRE sites identified as more highly enriched at 0, 12 and 36 hours were compared to the set of FAIRE sites that do not change using HOMER for the presence of known transcription factor binding motifs. Grey indicates a motif is enriched in the indicated group. For the bottom row, the set of differential FAIRE sites were pooled and compared as whole to the set of FAIRE sites that do not change (Orange indicates enrichment).

S3

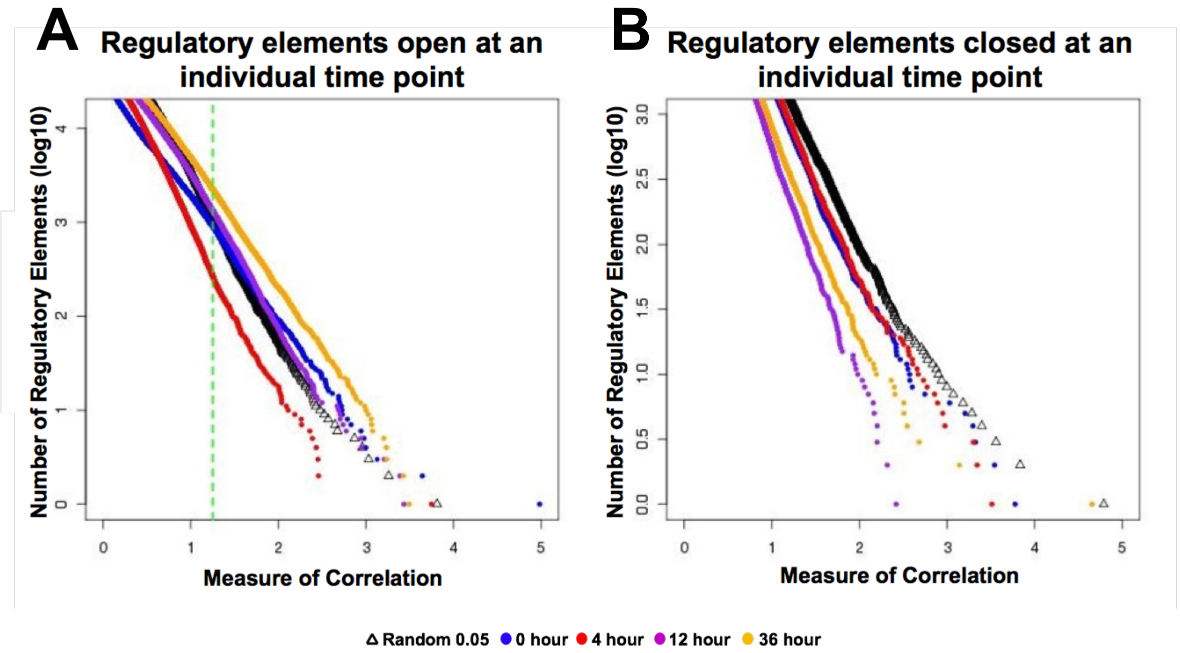

Supplementary Figure S3. Identification of differentially enriched FAIRE sites throughout the time-course. For each of the time-points, all three replicates were compared to the replicates for the other time-points to identify those sites with consistently different enrichment by FAIRE. The significance of these differences were assessed by permuting to randomly assign three different replicates to the comparison group, the black line represents 0.05. (A) FAIRE signal is higher at a given time-point and (B) FAIRE is lower in the given time-point.

S4

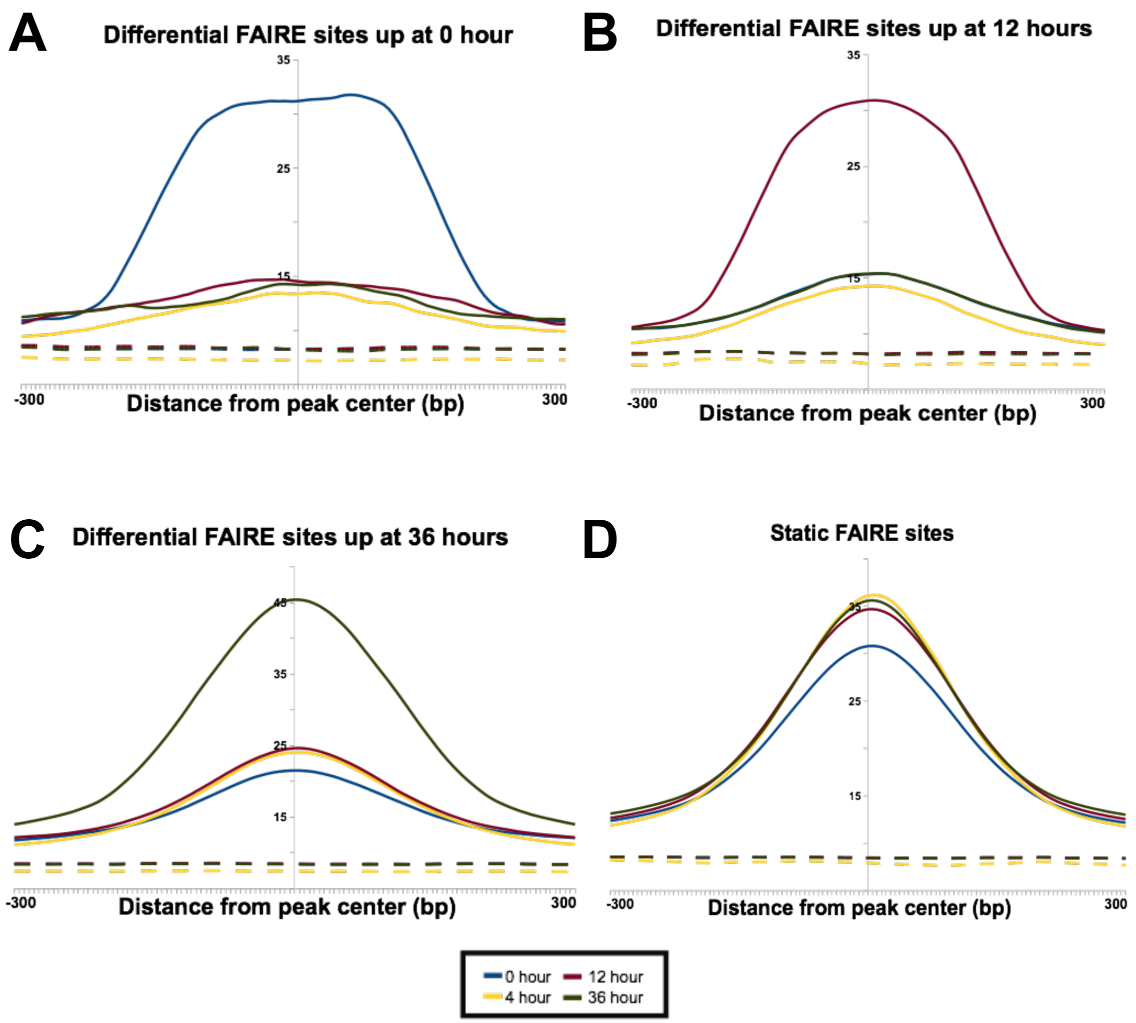

Supplementary Figure S4: Changes in FAIRE signal amplitude. **(A-D)** Shown is the normalized FAIRE signal for the set of regions within each of the respective groups compared to a set of regions randomly sampled throughout the genome (dashed lines, 10x more sites than those in group). The FAIRE signal for each of the respective groups is greater than the signal from the other time-points, but greater than the signal from background (or random).

S5

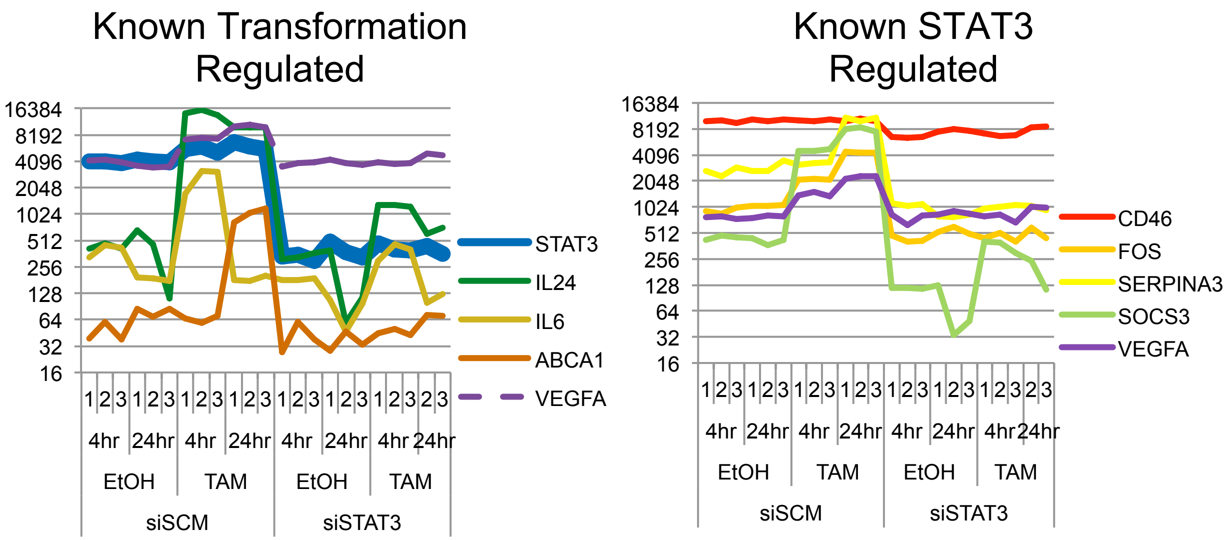

Supplementary Figure S5. **siSTAT3 knockdown and genes known to be regulated during transformation of MCF10A-ER-Src cells**

RNA expression values of STAT3 and four genes known to be differentially regulated during transformation of MCF10A-ER-Src cells (left). Genes known to be regulated by STAT3, indicating functional inactivation of STAT3 was achieved (right).

S6

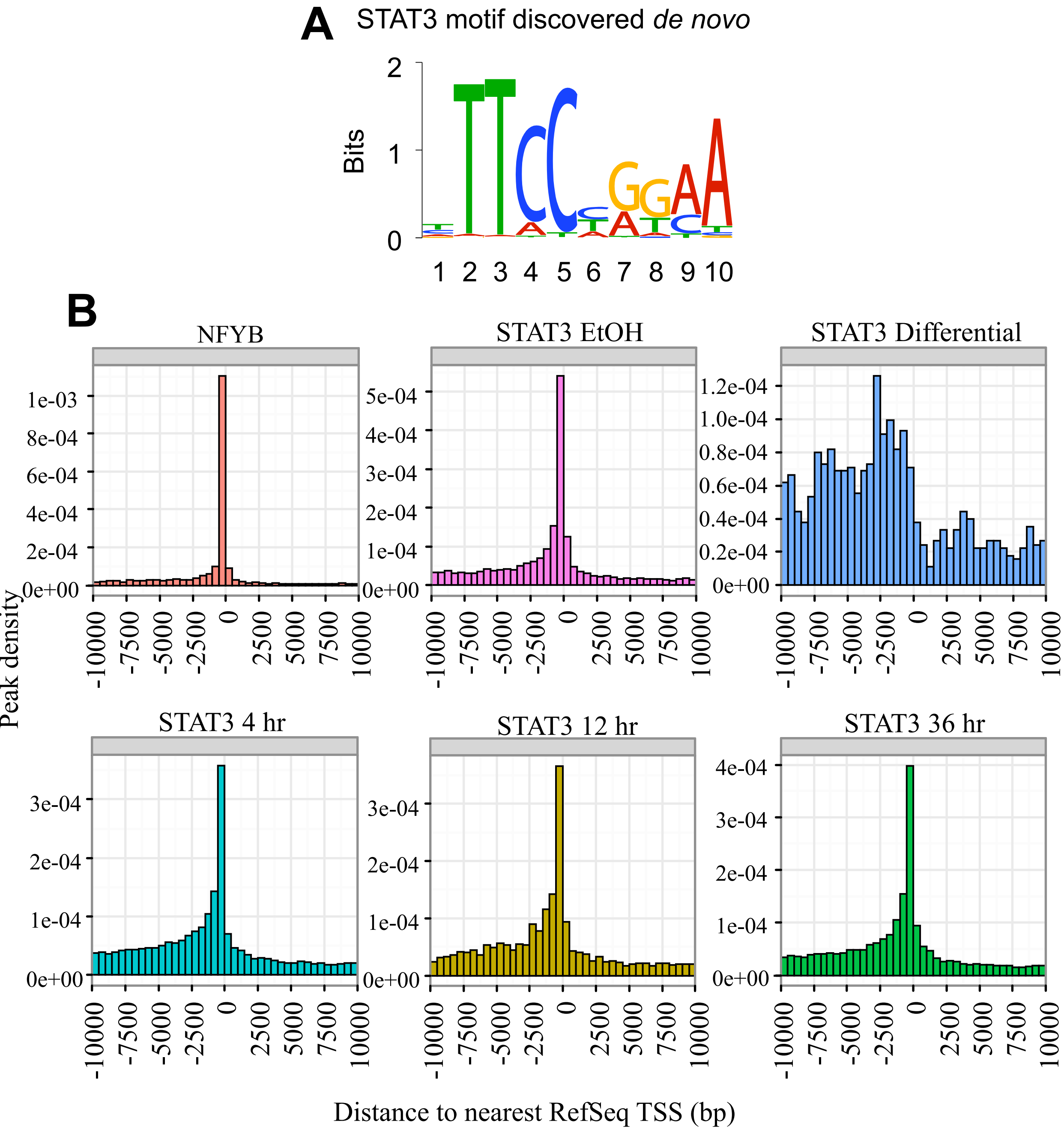

Supplementary Figure S6:Transformation induced differential STAT3 sites are preferentially located outside of proximal promoters. A. Canonical STAT3 motif discovered in top STAT3 ChIP-seq peaks. **B**. ChIP-Seq peak density of STAT3 and NF-Y around RefSeq TSSs. Differential STAT3 sites were located more distally than all STAT3 peaks from 4 hr, 12 hr and 36 hr post ER-Src induction. 0 bp represents the TSS.

S7

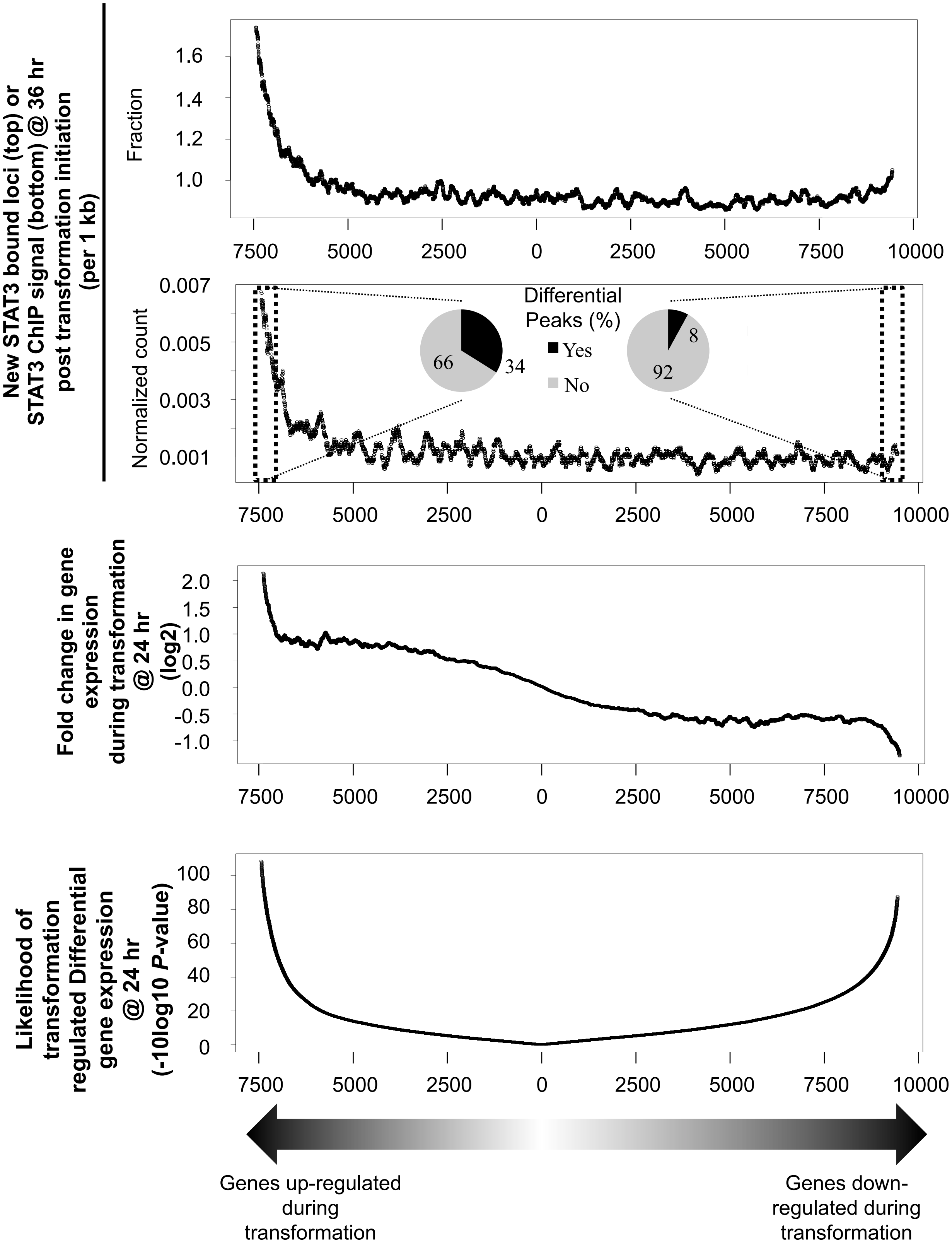

New STAT3 bound loci (top) or STAT3 ChIP signal (bottom) @ 36 hr post transformation initiation (per 1 kb)
Fraction
1.6
1.4
1.2
1.0
7500
5000
2500
0
2500
5000
7500
10000
Normalized count
0.007
0.005
0.003
0.001
Differential Peaks (%)
Yes
No
66
34
8
92
Fold change in gene expression during transformation @ 24 hr (log2)
2.0
1.5
1.0
0.5
0.0
-0.5
-1.0
Likelihood of transformation regulated Differential gene expression @ 24 hr (-10log10 P-value)
100
80
60
40
20
0
Genes up-regulated during transformation
Genes down-regulated during transformation

S7 Legend

Supplementary Figure S7: Association of transformation induced chromatin bound STAT3 with transformation-dependent differential gene expression. All genes differentially expressed during transformation were considered, separated into up- and down-regulated genes by siSTAT3 treatment, and sorted by the probability of differential gene regulation by siSTAT3. Plotted are: the number of transformation differential STAT3 loci (per kb, per region) that occurred at 24 hr post ER-Src induction within proximal promoter regions (+/- 2.5 kbp about TSS) or distal regions (+/- 50 kbp from TSS, excluding the proximal promoter region); and, the associated fold change in gene expression upon siSTAT3 treatment. Pie charts indicate the percentage of the top 500 regions that contained a differential STAT3 site at 4 hr post ER-Src induction.

S8

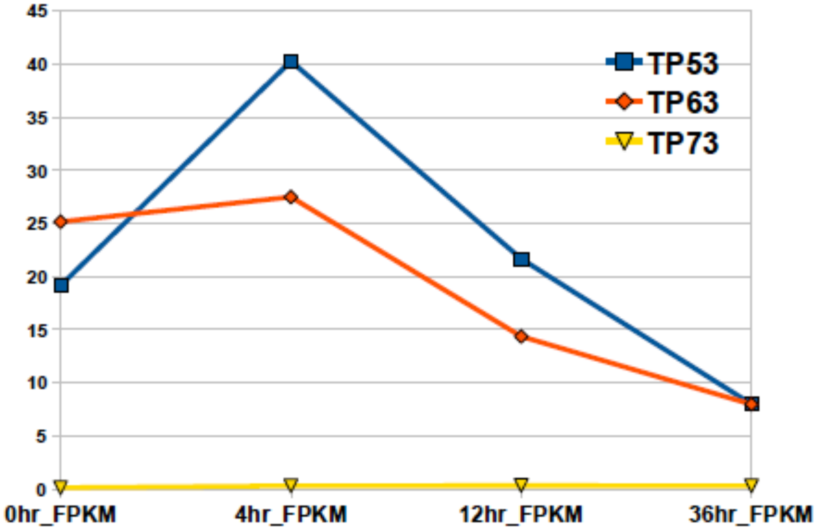

Supplementary Figure S8:RNA-seq levels of TP53, TP63 and TP73 post TAM Induction

S9A

Supplementary Figure S9: Gene ontology terms of STAT3 regulated genes during transformation. (A) Genes significantly differentially regulated during transformation that are also significantly affected by STAT3 knockdown. **(B)** Genes significantly differentially regulated during transformation that are not significantly affected by STAT3 knockdown

A

STAT3-dependent

4 hr		24 hr	
**Pathway**	***P*-value**	**Pathway**	***P*-value**
Acute Phase Response Signaling	4.0E-03	IL-6 Signaling	7.9E-08
Clathrin-mediated Endocytosis	6.2E-03	IL-10 Signaling	2.2E-07
IL-6 Signaling	6.3E-03	Biosynthesis of Steroids	2.6E-06
ILK Signaling	6.3E-03	PPAR Signaling	3.0E-06
IL-10 Signaling	9.1E-03	LXR/RXR Activation	3.5E-06
Role of Macrophages, Fibroblasts and Endothelial Cells in Rheumatoid	1.2E-02	Acute Phase Response Signaling	5.5E-06
IL-9 Signaling	1.9E-02	Hepatic Fibrosis / Hepatic Stellate Cell Activation	6.5E-06
Oncostatin M Signaling	1.9E-02	LPS/IL-1 Mediated Inhibition of RXR Function	9.1E-06
IL-17A Signaling in Fibroblasts	2.0E-02	Role of Macrophages, Fibroblasts and Endothelial Cells in Rheumatoid Arthritis	4.1E-05
p53 Signaling	2.1E-02	Oncostatin M Signaling	2.5E-04
Atherosclerosis Signaling	3.9E-02	Hepatic Cholestasis	2.8E-04
LXR/RXR Activation	4.2E-02	PPAR/RXR Activation	2.8E-04
LPS/IL-1 Mediated Inhibition of RXR Function	4.8E-02	Cholecystokinin/Gastrin-mediated Signaling	4.7E-04
IL-12 Signaling and Production in Macrophages	5.2E-02	Sertoli Cell-Sertoli Cell Junction Signaling	4.8E-04
Erythropoietin Signaling	6.8E-02	VDR/RXR Activation	7.4E-04
JAK/Stat Signaling	6.8E-02	Type I Diabetes Mellitus Signaling	9.3E-04
Growth Hormone Signaling	6.9E-02	IGF-1 Signaling	1.3E-03
Renal Cell Carcinoma Signaling	7.2E-02	p38 MAPK Signaling	1.5E-03
Prolactin Signaling	7.8E-02	Graft-versus-Host Disease Signaling	1.7E-03
VEGF Family Ligand-Receptor Interactions	8.3E-02	Role of Osteoblasts, Osteoclasts and Chondrocytes in Rheumatoid Arthritis	1.8E-03

S9B

B

STAT3-independent

4 hr		24 hr	
**Pathway**	***P*-value**	**Pathway**	***P*-value**
IL-12 Signaling and Production in Macrophages	1.9E-04	Butanoate Metabolism	1.6E-03
Activation of IRF by Cytosolic Pattern Recognition Receptors	2.5E-04	Complement System	5.2E-03
Pancreatic Adenocarcinoma Signaling	3.8E-04	Bile Acid Biosynthesis	5.9E-03
iNOS Signaling	6.3E-04	IL-8 Signaling	6.2E-03
PI3K/AKT Signaling	6.6E-04	Androgen and Estrogen Metabolism	6.6E-03
Colorectal Cancer Metastasis Signaling	1.3E-03	Actin Nucleation by ARP-WASP Complex	6.9E-03
Role of IL-17A in Arthritis	1.4E-03	Tumoricidal Function of Hepatic Natural Killer Cells	1.4E-02
p53 Signaling	1.5E-03	Pentose and Glucuronate Interconversions	1.4E-02
Chronic Myeloid Leukemia Signaling	1.5E-03	Starch and Sucrose Metabolism	1.4E-02
Hepatic Fibrosis / Hepatic Stellate Cell Activation	1.6E-03	p53 Signaling	1.5E-02
Role of Pattern Recognition Receptors in Recognition of Bacteria and Viruses	1.7E-03	NRF2-mediated Oxidative Stress Response	1.5E-02
TNFR2 Signaling	2.1E-03	Galactose Metabolism	1.7E-02
CD40 Signaling	2.5E-03	PDGF Signaling	1.8E-02
Retinoic acid Mediated Apoptosis Signaling	2.5E-03	HGF Signaling	2.0E-02
Inositol Phosphate Metabolism	2.7E-03	Aminosugars Metabolism	2.0E-02
Type I Diabetes Mellitus Signaling	3.0E-03	Arginine and Proline Metabolism	2.0E-02
PTEN Signaling	3.3E-03	Cholecystokinin/Gastrin-mediated Signaling	2.1E-02
Interferon Signaling	3.4E-03	Role of Tissue Factor in Cancer	3.0E-02
Molecular Mechanisms of Cancer	3.5E-03	Estrogen-Dependent Breast Cancer Signaling	4.0E-02
Small Cell Lung Cancer Signaling	3.7E-03	Extrinsic Prothrombin Activation Pathway	4.5E-02

Supplementary Figure S10: Ingenuity pathway analysis of genes differentially regulated during transformation. Lines between two genes indicate a known or predicted, direct or indirect interaction. See legend for details. **(A)** The top interaction network derived from STAT3 dependent differentially regulated genes at 4 hr post induction of ER-Src. Green and red shading indicate down and up regulated by siSTAT3 treatment, respectively. Only genes that are differentially regulated by transformation and by siSTAT3 are considered. (**B)** Similar to A except at 24 hr post ER-Src induction. **(C)** The top interaction network derived from STAT3 independent differentially regulated genes at 4 hr post induction of ER-Src. Green and red shading indicate down and up regulated during transformation, respectively. Only genes that are differentially regulated by transformation and not by siSTAT3 are considered. (D) Similar to C except at 24 hr post ER-Src induction.

A

Pathway: Organismal Injury and Abnormalities, Cellular Movement, Nervous System Development and Function

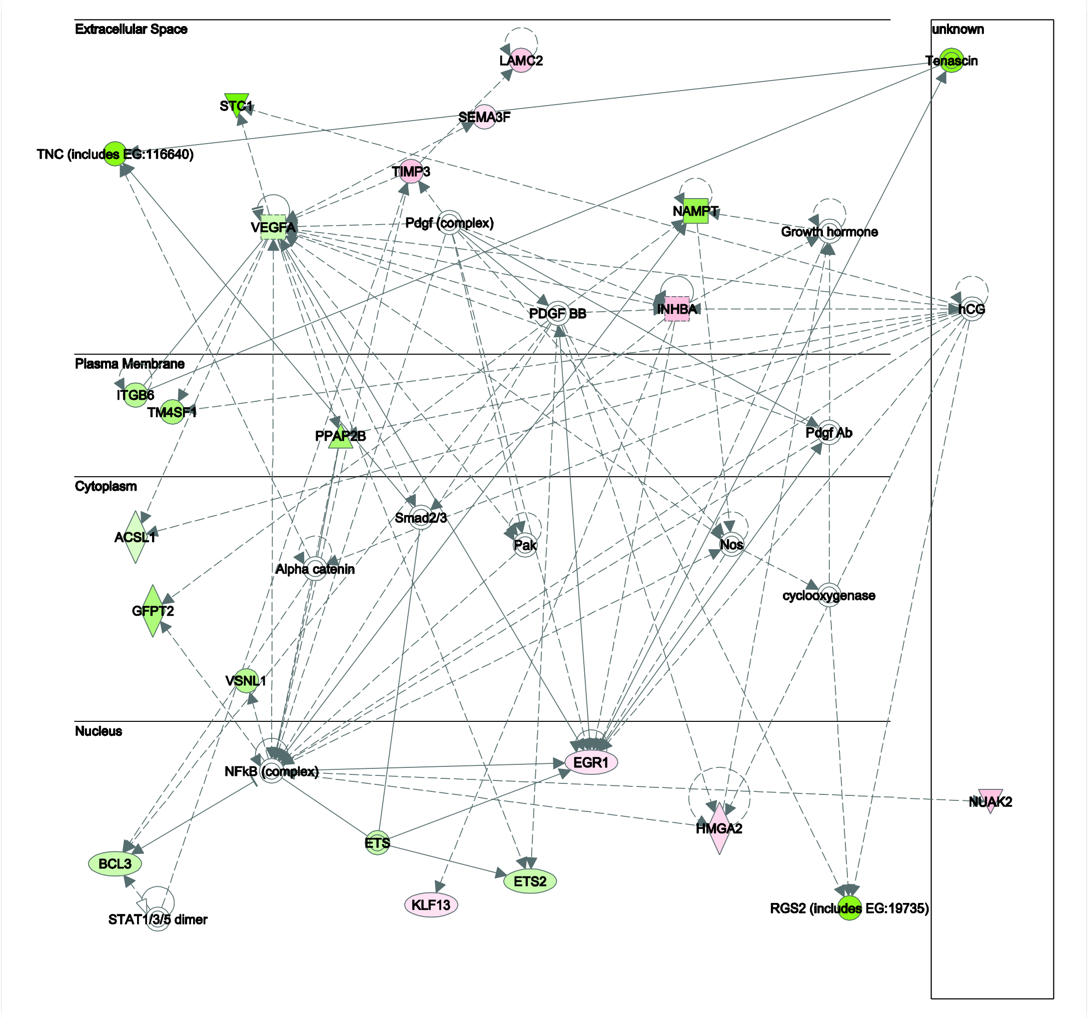

© 2000-2012 Ingenuity Systems, Inc. All rights reserved.

S10B

B

Transformation- and STAT3-dependent differentially expressed genes – 24 hr

Pathway: Cellular Assembly and Organization, Embryonic Development, Organ Development

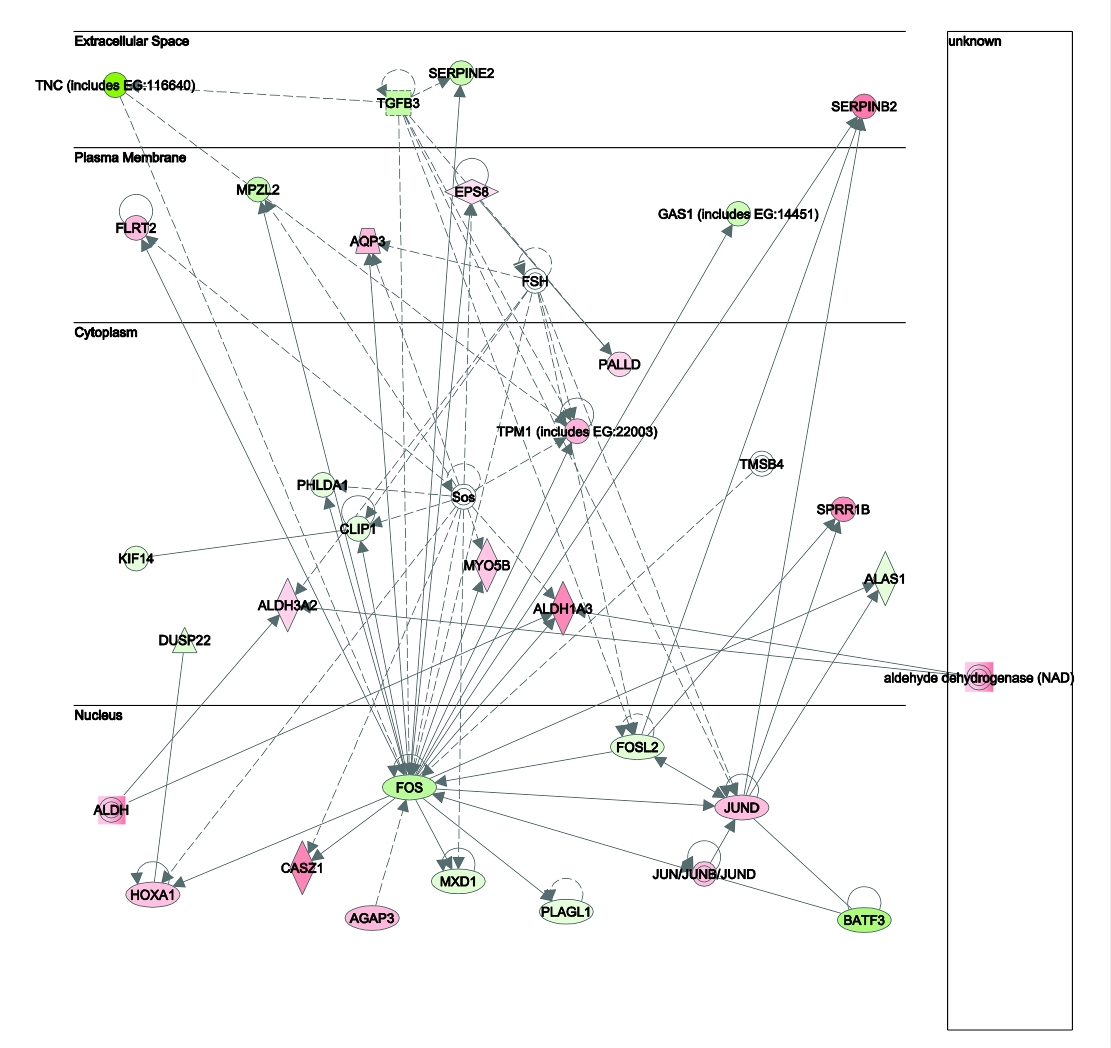

© 2000-2012 Ingenuity Systems, Inc. All rights reserved.

S10C

C Transformation-dependent and STAT3-independent differentially expressed genes – 4 hr

Pathway: Cancer, Hereditary Disorder, Reproductive System Disease

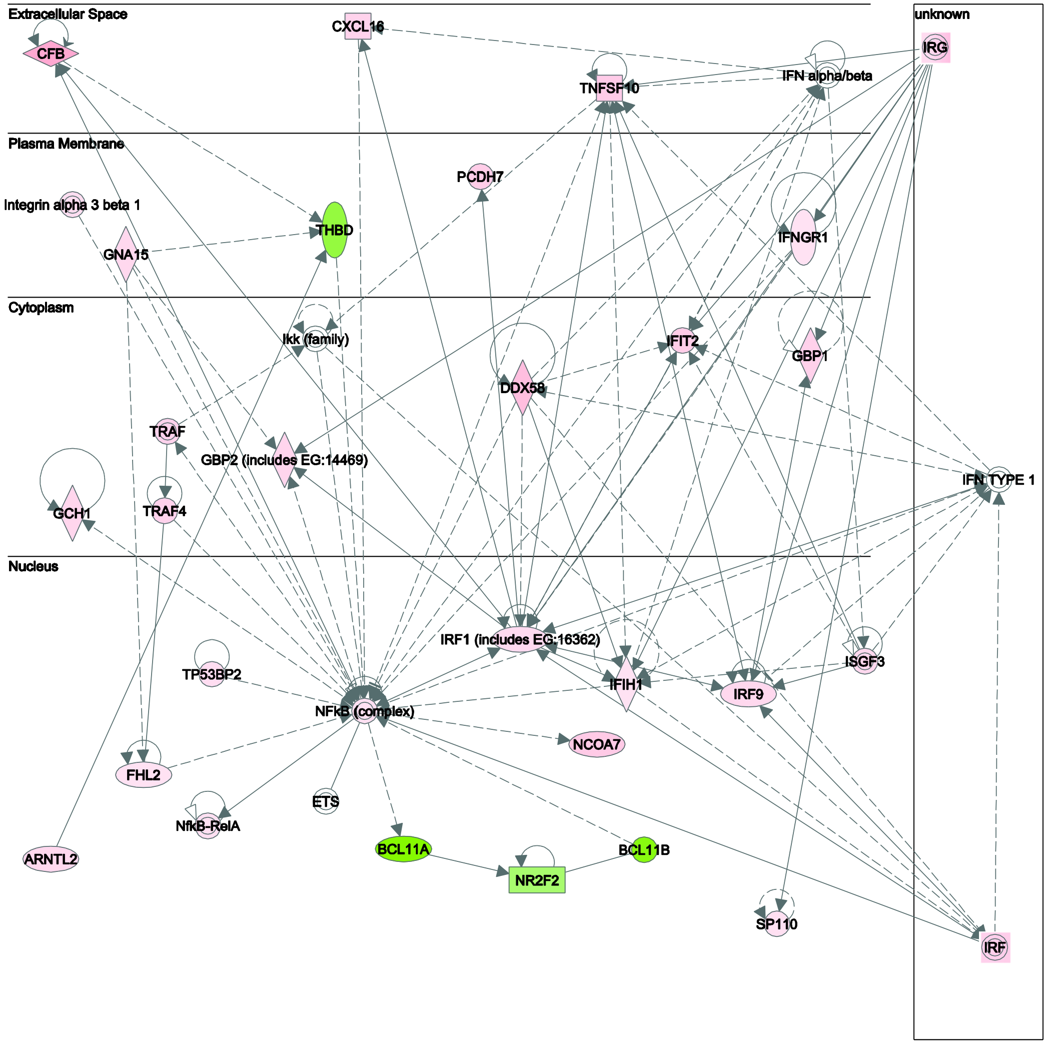

© 2000-2012 Ingenuity Systems, Inc. All rights reserved.

S10
D

D Transformation-dependent and STAT3-independent differentially expressed genes – 24 hr

Pathway: Carbohydrate Metabolism, Drug Metabolism, Small Molecule Biochemistry

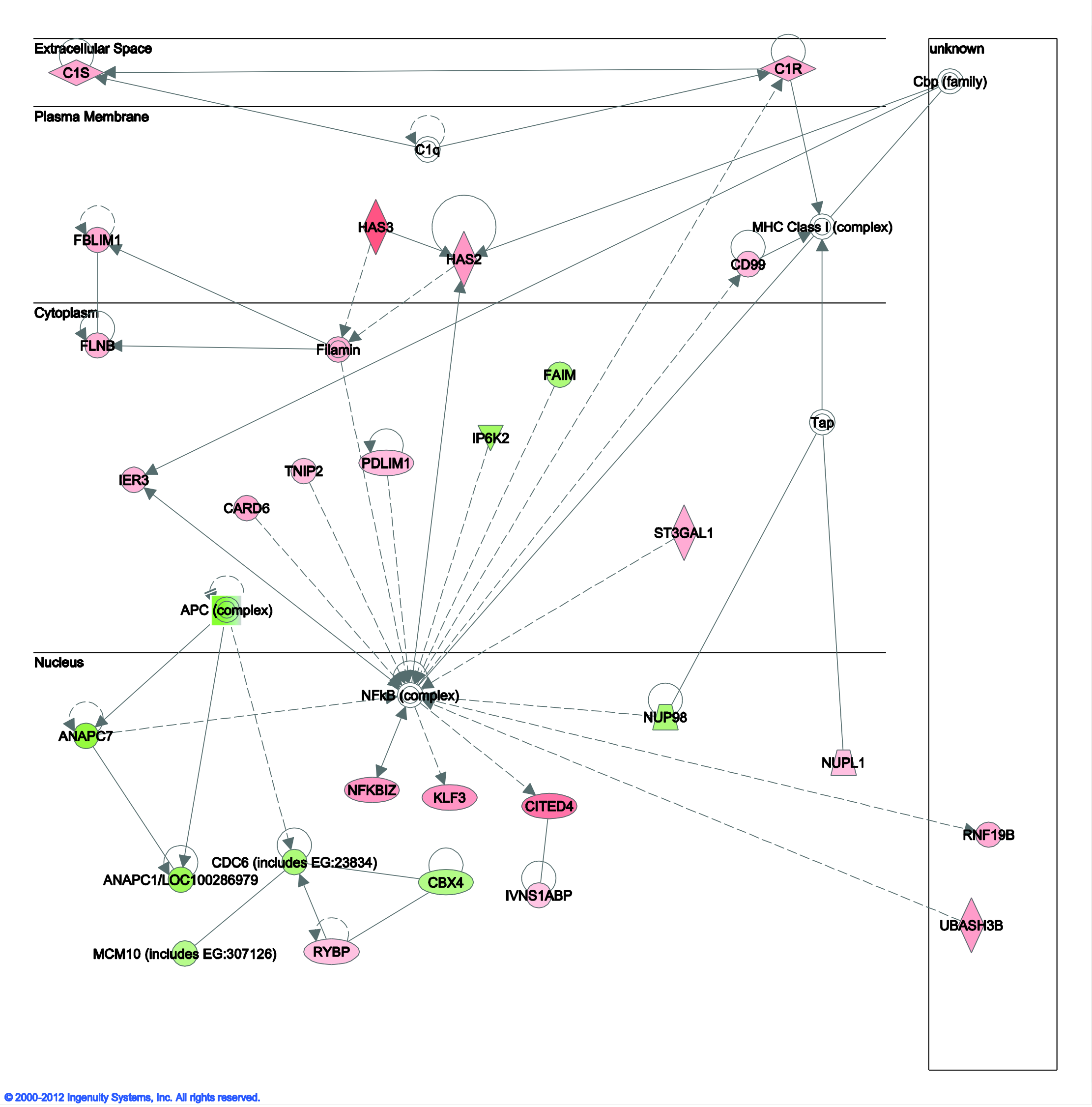

© 2000-2012 Ingenuity Systems, Inc. All rights reserved.

Supplemental Figure 10 Symbol Legends

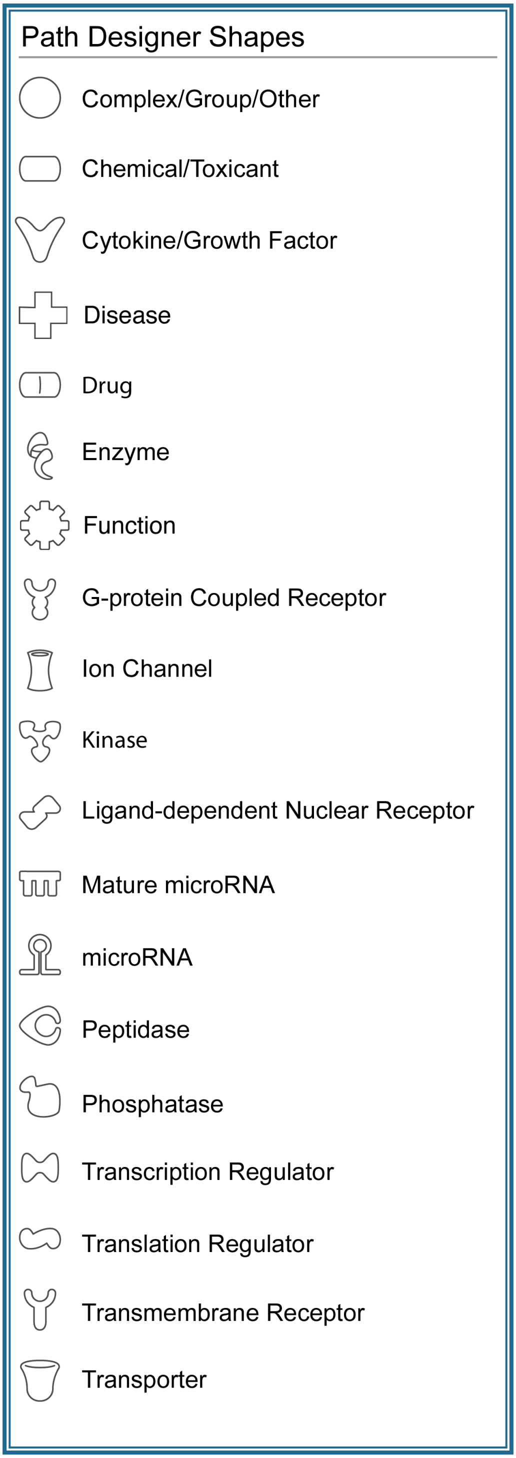

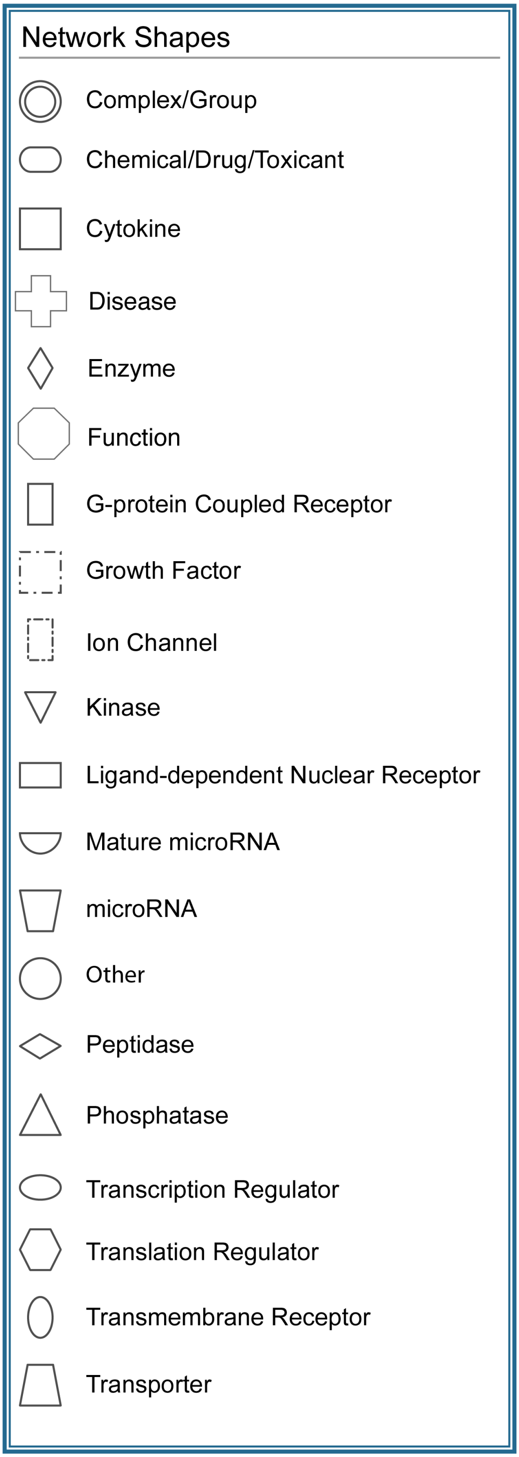

Supplemental Figure 10 Symbol Legends, continued

Relationships

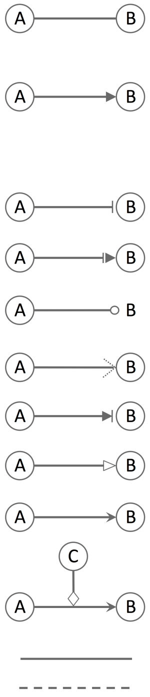

- chemical-chemical interactions, chemical-protein interactions, correlation, protein-protein interactions, RNA-RNA interactions: non targeting interactions
- activation, causation, expression, localization, membership, modification, molecular cleavage, phosphorylation, protein-DNA interactions, protein-RNA interactions, regulation of binding, transcription
- inhibition, ubiquitination
- inhibits and acts on
- leads to
- processing yields
- RNA-RNA interactions: microRNA targeting
- translocation
- reaction
- enzyme catalysis
- direct interaction
- indirect interaction

Relationship Labels

A	Activation
B	Binding
C	Causation/Leads to
CO	Correlation
CC	Chemical-Chemical interaction
CP	Chemical-Protein interaction
E	Expression (includes metabolism/ synthesis for chemicals)
EC	Enzyme Catalysis
I	Inhibition
L	Proteolysis (includes degradation for Chemicals)
LO	Localization
M	Biochemical Modification
miT	microRNA Targeting
MB	Group/complex Membership
nTRR	Non-Targeting RNA-RNA Interaction
P	Phosphorylation/Dephosphorylation
PD	Protein-DNA binding
PP	Protein-Protein binding
PR	Protein-RNA binding
PY	Processing Yields
RB	Regulation of Binding
RE	Reaction
RR	RNA-RNA Binding
T	Transcription
TR	Translocation
UB	Ubiquitination

S11

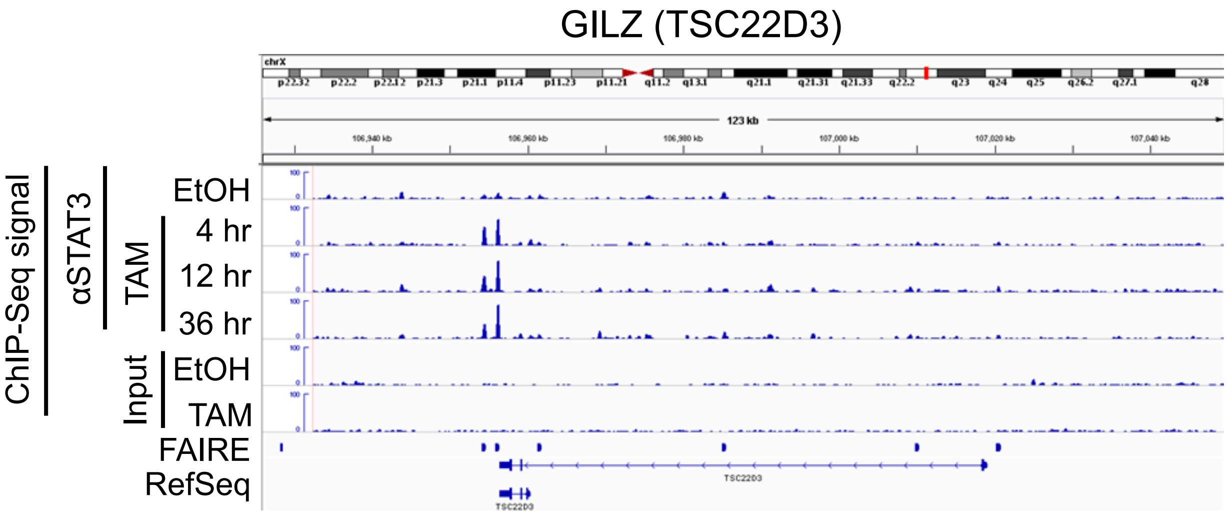

Supplementary Figure S11. Localization of STAT3 occupancy at the GILZ genomic locus.
Raw ChIP-seq signal of STAT3 during tamoxifen induced transformation of MCF10A-ER-Src cells.

S12

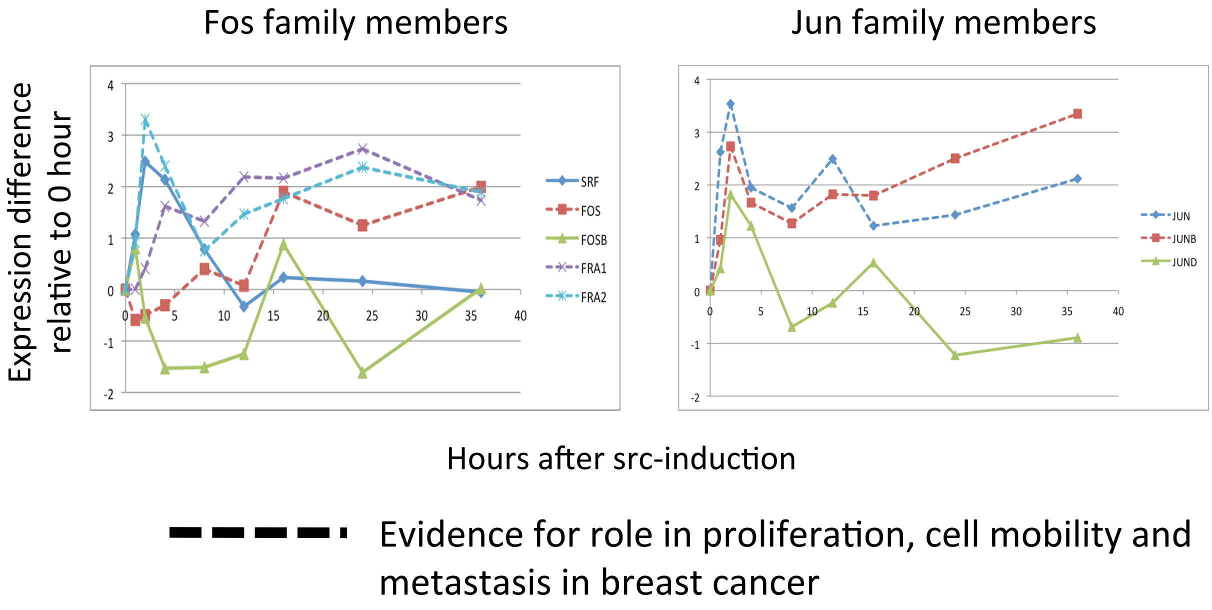

Supplementary Figure S12. **Members of the AP-1 complex are differentially regulated throughout the transformation.** Transcription of Fos and Jun family members implicated in carcinogenesis is upregulated during cellular transformation.
